# Expression of Concern: Effects of High and Low Fat Dairy Food on Cardio-Metabolic Risk Factors: A Meta-Analysis of Randomized Studies

**DOI:** 10.1371/journal.pone.0283275

**Published:** 2023-11-13

**Authors:** The PLOS ONE Editors

Concerns were raised about repeated mean and standard deviation values reported in this article’s figures [1]. In reviewing this matter, the PLOS Editors noted several discrepancies between the mean and standard deviation values reported in the *PLOS ONE* article figures and the corresponding results reported in the underlying primary literature.

In response to these concerns, the corresponding author re-reviewed the data reported in [1] versus the primary literature and found that there had been numerous data extraction, recording, or reporting errors. The corrected dataset in S1 File of this notice was used to repeat the forest plot and funnel plot analyses. See Figs 2–10 of the republished article for the updated forest plots. The funnel plots are in S2 File of this notice. A statistical reviewer reviewed the updates and confirmed that the main results and conclusions are supported by the revised analyses.

The authors also provided the following clarifications and updates:

- In cases where neither standard deviation (SD) nor standard error (SE) were reported in the primary literature, the weighted mean SD for the relevant outcome was used as noted in the text below the updated figures. Weighted mean SD values were calculated based on the SD values reported for the indicated outcome in other included studies, as shown in S1 File. In light of this issue, the updated figures report some repeat SD values in cases where SD and SE values were not available for multiple studies and the dataset’s weighted mean SD was used.
- In cases where the primary literature reported SE or confidence intervals (CI) instead of SD, RevMan software was used to calculate SD based on the SE or CI values. S1 File indicates where such conversions were required.
- Table footnotes in Stancliffe (2011) indicated that the table reported mean +/- SD; the authors of [1] hypothesized that the SD values were labelled incorrectly in the 2011 article and instead represented SE. If Stancliffe (2011) correctly labelled these results, the SD values are outliers as compared to other studies; if there was a reporting error and these are instead SE values, the results are consisted with other studies. The SD values reported for Stancliffe (2011) in the updated figures (S2 File) were obtained by converting the values reported in Stancliffe (2011) using an SE to SD conversion tool.
- Stancliffe (2011) was removed from the LDL cholesterol table in the revised figures (S2 File) because the original article reported oxidized cholesterol, which differs from the measure used in the other studies.
- In Figure 8 in [1] the N value was incorrectly reported for the Chrichton study. This has been corrected in the updated figure.
- The article reports conclusions based on funnel plots and Begg’s tests, but these results were not included with the article or its supporting information. S2 File includes funnel plots for the reanalyses reported in this notice, but Begg’s test results were not provided.

Whilst mean differences and confidence intervals have shifted with these corrections, the changes have not impacted the overall conclusion of the study that increased dairy food intake (particularly low fat dairy food intake) is associated with increased weight but has no significant effects on other cardiometabolic risk factors.

There were several figure citation errors in the original Results section, which have been corrected in the republished version.

In addition, there were reference errors in [1]:

- References 45 and 67 in [1] represent the same article and lack citation details. This article was published in 2014 and is incorrectly cited in [1] as a 2013 article. The correct reference is:Benatar JR, Jones E, White H, Stewart RA. A randomized trial evaluating the effects of change in dairy food consumption on cardio-metabolic risk factors. Eur J Prev Cardiol. 2014 Nov;21(11):1376–86. doi: 10.1177/2047487313493567. Epub 2013 Jun 17. PMID: 23774272.
- The publication year is incorrectly reported in Reference 42 and the citations within the article and figures. The article was published in 2011 and the correct reference is:
- Stancliffe RA, Thorpe T, Zemel MB. Dairy attentuates oxidative and inflammatory stress in metabolic syndrome. Am J Clin Nutr. 2011 Aug;94(2):422–30. doi: 10.3945/ajcn.111.013342. Epub 2011 Jun 29. PMID: 21715516; PMCID: PMC3142721.

The article [1] was republished on November 13, 2023 to provide updated Figs 2–10 and Tables 1 and 2, correct the values reported in the text for these analyses, and address the other reporting errors described above. Please download the new version of the article PDF. For transparency, the original article’s PDF is attached to this notice as S3 File, and a tracked changes version of the manuscript showing changes made at the time of republication is in S4 File.

In reviewing the updated submission files, PLOS noted discrepancies between the data file (S1 File) and some of the results reported in the revised figures. Most but not all issues were resolved prior to the republication.

The *PLOS ONE* Editors issue this Expression of Concern to notify readers above the issues.

## Supporting information

S1 File Excel file with amended datasets. (XLSX)

S2 File Funnel plot results based on analyses of the amended datasets. (DOCX)

S3 File Original published version of the article (PDF). (PDF)

S4 File Tracked changes version comparing the original published version of the article to the republished version. (DOCX)
