# Supplementary material for: Effects of High and Low Fat Dairy Food on Cardio-Metabolic Risk Factors: A Meta-Analysis of Randomized Studies
Source: PLoS One. 2013 Oct 11;8(10):e76480. doi: 10.1371/journal.pone.0076480 (PMC3795726; doi:10.1371/journal.pone.0076480)
Supplement: File S1 — Prisma flow chart. (PDF) [file pone.0076480.s002.pdf]

PRISMA 2009 Flow Diagram

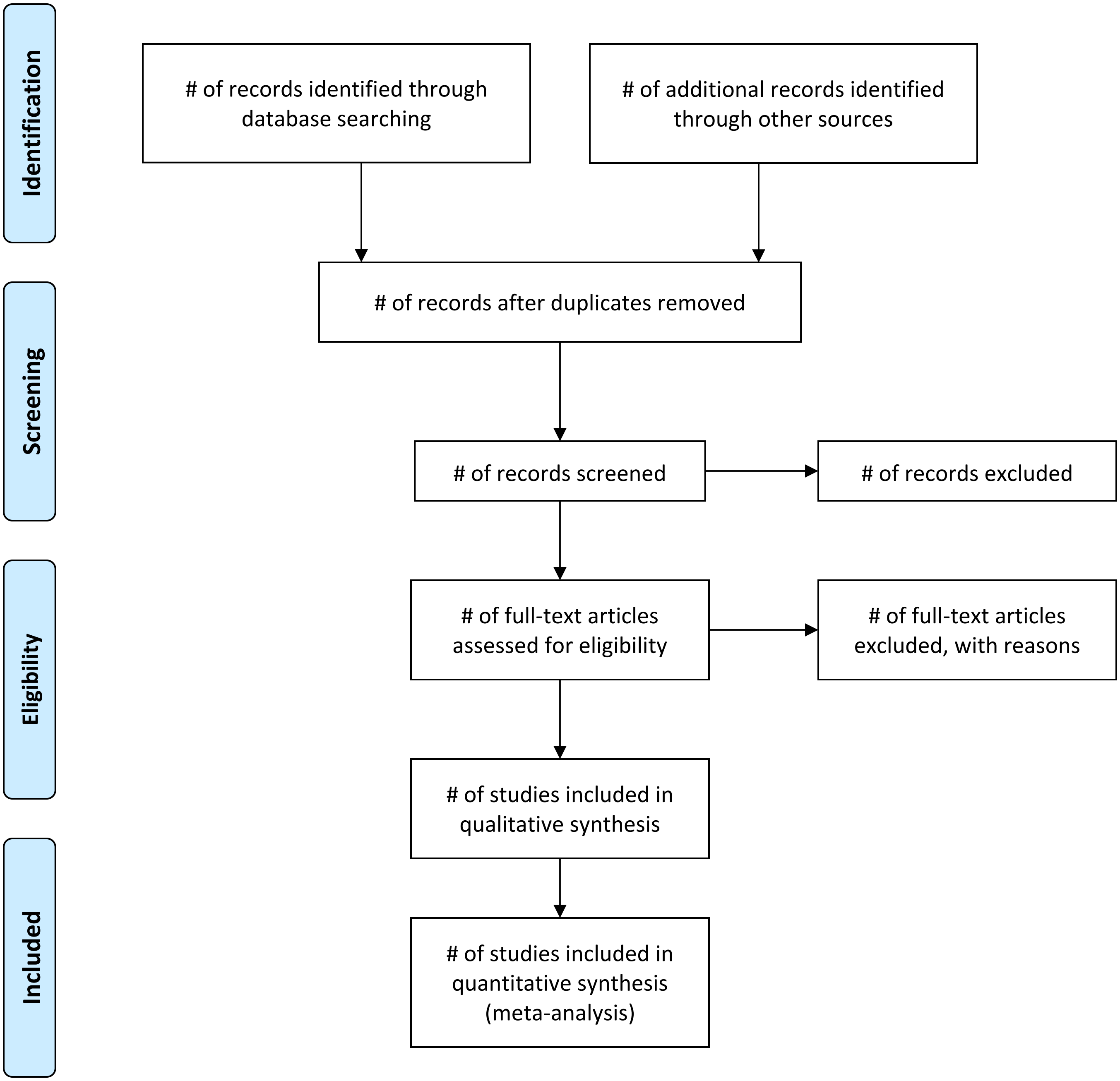

From: Moher D, Liberati A, Tetzlaff J, Altman DG, The PRISMA Group (2009). *P*referred *R*eporting *I*tems for *S*ystematic Reviews and *M*eta-*A*nalyses: The PRISMA Statement. PLoS Med 6(6): e1000097. doi:10.1371/journal.pmed1000097

For more information, visit www.prisma-statement.org.
