# Supplementary material for: Effects of High and Low Fat Dairy Food on Cardio-Metabolic Risk Factors: A Meta-Analysis of Randomized Studies
Source: PLoS One. 2013 Oct 11;8(10):e76480. doi: 10.1371/journal.pone.0076480 (PMC3795726; doi:10.1371/journal.pone.0076480)
Supplement: File S2 — Protocol. (DOCX) [file pone.0076480.s003.docx]

Effects of high and low fat dairy food on cardio-metabolic risk factors:

A meta-analysis of randomized studies

Study protocol

Jocelyne Benatar

# Background

Diary food especially low fat dairy food is considered to improve cardio metabolic risk factors such as weight, blood pressure ad insulin resistance. This is based on observational studies that show increased dairy food consumption is associated with less weight gain[^1^](#_ENREF_1), reduced risk of diabetes[^2^](#_ENREF_2)^,^ [^3^](#_ENREF_3) and lower blood pressure[^4^](#_ENREF_4). Dairy food is the main source of saturated fats that are thought to increase cardiovascular risk and adversely affect lipids[^5^](#_ENREF_5). Subsequently most food guidelines recommend daily consumption of low fat dairy food so that consumers receive the ‘health benefits’ of dairy calcium and protein but avoid adverse health effects of saturated fats. These guidelines are however not based on evidence

Dairy is a complex food which contains short, medium and long chain saturated fats which may have differing health effects[^6^](#_ENREF_6). Observational studies assessing dairy food intake are confounded by multiple factors for example dairy food intake is positively associated with healthier lifestyle behaviors and higher socio economic status[^7^](#_ENREF_7). Large randomized studies[^8^](#_ENREF_8)^,^ [^9^](#_ENREF_9) assessing the effects of dietary patterns on cardiovascular health have not focused on the effects of dairy food. The effects of dairy food on cardio metabolic risk factors have been assessed in multiple small studies. A recent meta-analysis[^10^](#_ENREF_10) has assessed the effects of all dairy food on weight but did not compare effects of whole and low fat dairy food. No meta analysis has assessed effects on other cardio metabolic risk factors.

Aim: To evaluate the effects of increased dairy food in a healthy population on cardio metabolic risk factors.

Objective: To see effects of increasing dairy on blood pressure, HOMA, weight, waist circumference, lipids, C- reactive protein

Methods: A meta- analysis using Rev Man 5 software assessing the effects of dietary interventions to increase dairy intake for at least on month will be performed. No date restriction will be applied. All randomized control studies with dairy as intervention that reported any of the outcome measures in healthy populations is eligible. All databases (science direct, Google, Cochrane database, Medline) will be searched.

### Inclusion criteria

1. Randomized controlled study
2. Participants > 18 years of age
3. Intervention longer than 1 month
4. Control arm is usual diet or reduced dairy food
5. Increased whole or low fat dairy food

### Exclusion Criteria

1. Observational or not randomized
2. Participants have diabetes, hypertension, heart disease
3. Participants on medications for lipids or blood pressure
4. Caloric restriction or other dietary intervention
5. Control arm is another intervention

Data extraction: 2 people will assess all articles for inclusion and exclusion criteria. Baseline characteristics of the participants, the intervention, effects on any cardio metabolic risk factor and funding source will be obtained. Each study will be scored using Jadad score. If any data is missing, corresponding authors will be contacted. All data will be converted to SI units and mean change and standard deviations will be used for analysis. Three people will then review every included study to ensure they meet inclusion and exclusion criteria. Every study will be assessed for study design and risk of bias.

Statistics Cases will be compared only with controls within the same study. For those studies with 3 treatment groups, comparison will be made with control and the high dairy group. Differences by dietary intervention are calculated as weighted mean difference divided by variance, expressed as a sample weighted mean effect size (95% confidence interval). A test for homogeneity will be performed for each cardio metabolic risk evaluated. Stratified analysis will be performed if there is enough data.

Ethics approval: Ethics approval for the study will be sought prior to any study procedure.

# References

1. Snijder MB, van der Heijden AA, van Dam RM, et al. Is higher dairy consumption associated with lower body weight and fewer metabolic disturbances? The Hoorn Study. *The American journal of clinical nutrition* 2007; **85**(4): 989-95.

2. Choi HK, Willett WC, Stampfer MJ, Rimm E, Hu FB. Dairy consumption and risk of type 2 diabetes mellitus in men: a prospective study. *Archives of internal medicine* 2005; **165**(9): 997-1003.

3. Elwood PC, Pickering JE, Fehily AM. Milk and dairy consumption, diabetes and the metabolic syndrome: the Caerphilly prospective study. *Journal of epidemiology and community health* 2007; **61**(8): 695-8.

4. Engberink MF, Geleijnse JM, de Jong N, Smit HA, Kok FJ, Verschuren WM. Dairy intake, blood pressure, and incident hypertension in a general Dutch population. *The Journal of nutrition* 2009; **139**(3): 582-7.

5. Reduced or modified dietary fat for preventing cardiovascular disease. *Cochrane Database Syst Rev* 2011; (7): CD002137. doi: 10.1002/14651858.CD002137.pub2.

6. Mansson HL. Fatty acids in bovine milk fat. *Food & nutrition research* 2008; **52**.

7. Parsons TJ, Power C, Logan S, Summerbell CD. Childhood predictors of adult obesity: a systematic review. *International journal of obesity and related metabolic disorders : journal of the International Association for the Study of Obesity* 1999; **23 Suppl 8**: S1-107.

8. Estruch R, Ros E, Salas-Salvado J, et al. Primary Prevention of Cardiovascular Disease with a Mediterranean Diet. *The New England journal of medicine* 2013.

9. Appel LJ, Moore TJ, Obarzanek E, et al. A clinical trial of the effects of dietary patterns on blood pressure. DASH Collaborative Research Group. *The New England journal of medicine* 1997; **336**(16): 1117-24.

10. Chen M, Pan A, Malik VS, Hu FB. Effects of dairy intake on body weight and fat: a meta-analysis of randomized controlled trials. *The American journal of clinical nutrition* 2012; **96**(4): 735-47.
