# Supplementary material for: Expression of Concern: Effects of High and Low Fat Dairy Food on Cardio-Metabolic Risk Factors: A Meta-Analysis of Randomized Studies
Source: PLoS One. 2023 Nov 13;18(11):e0283275. doi: 10.1371/journal.pone.0283275 (PMC10642843; doi:10.1371/journal.pone.0283275)
Supplement: S2 File — (DOCX) [file pone.0283275.s002.docx]

S2 File [File legend] Funnel plot results based on analyses of the amended datasets

In the funnel plots of effects of fat dairy food on waist circumference, HOMA-IR, glucose, LDL-c, HDL-c, C - reactive protein, systolic and diastolic blood pressure. These funnel plots indicate that the most common outliers is the study from Stancliffe et al.


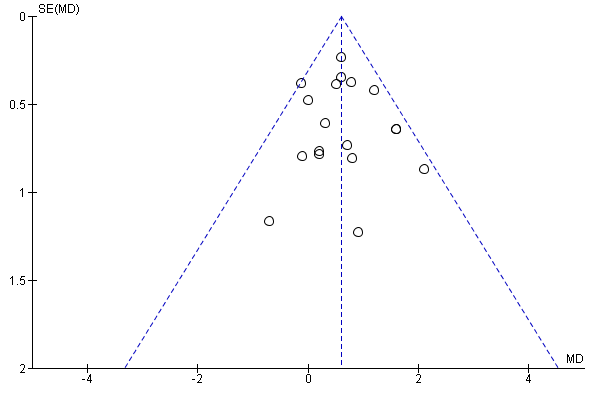


Weight


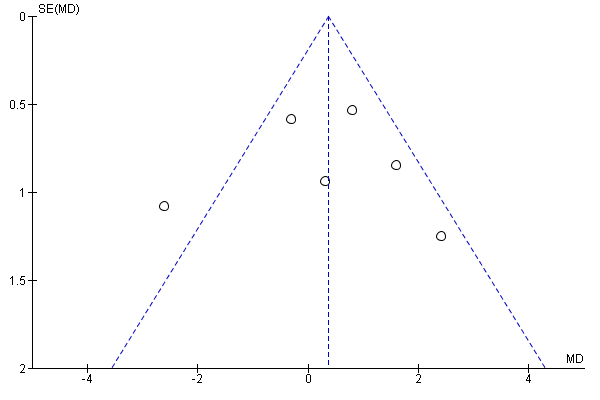


Waist circumference


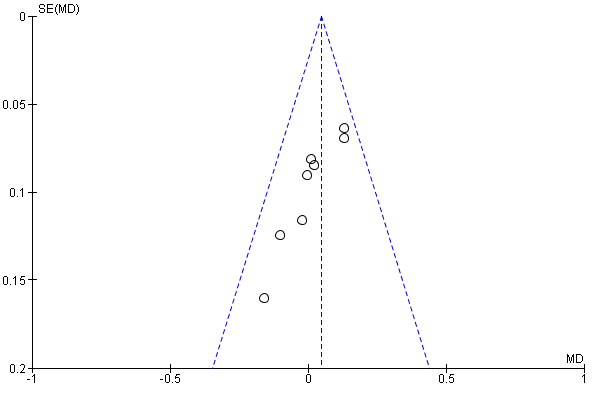


Blood glucose


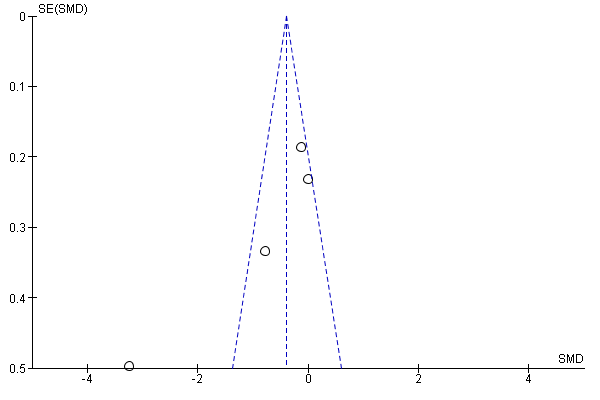


HOMA- IR


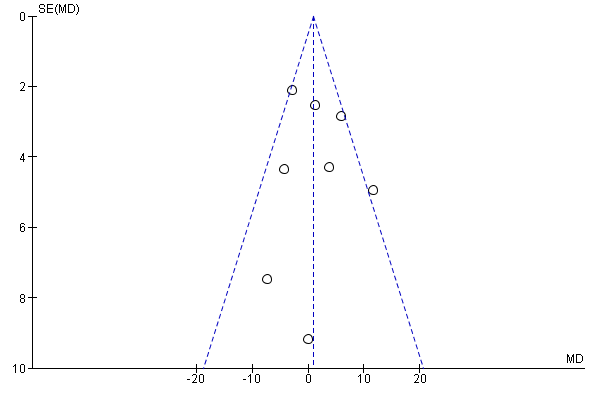


HDL-c

LDL-c


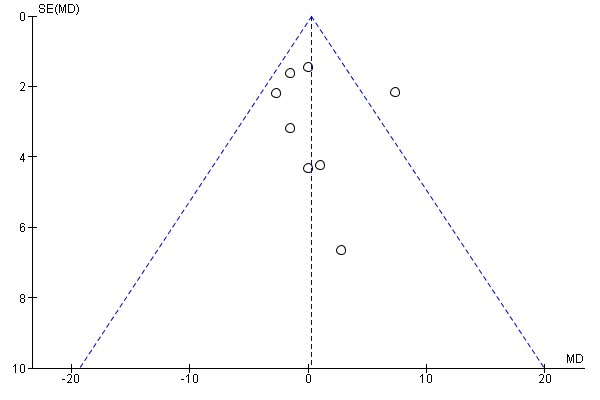


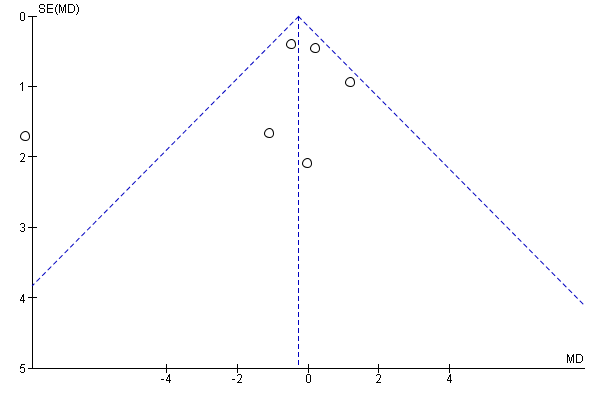


CRP


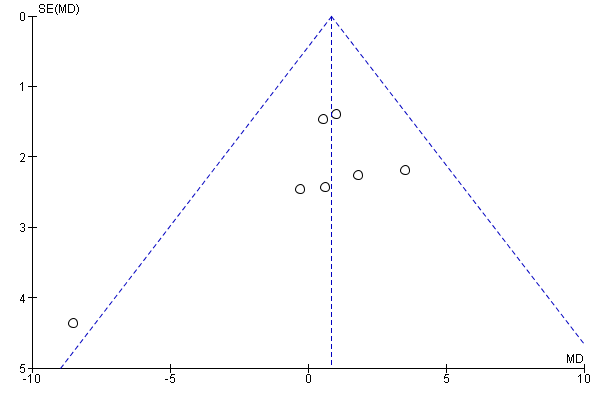


Systolic BP


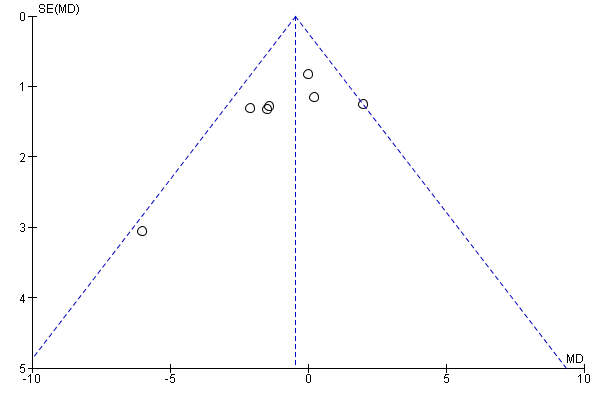


Diastolic BP
