# Supplementary material for: Expression of Concern: Effects of High and Low Fat Dairy Food on Cardio-Metabolic Risk Factors: A Meta-Analysis of Randomized Studies
Source: PLoS One. 2023 Nov 13;18(11):e0283275. doi: 10.1371/journal.pone.0283275 (PMC10642843; doi:10.1371/journal.pone.0283275)
Supplement: S3 File — (PDF) [file pone.0283275.s003.pdf]

OPEN ACCESS Freely available online

Effects of High and Low Fat Dairy Food on Cardio-Metabolic Risk Factors: A Meta-Analysis of Randomized Studies

Jocelyne R. Benatar*, **Karishma Sidhu**, **Ralph A. H. Stewart**

Green Lane Cardiovascular Service, Auckland City Hospital, Auckland, New Zealand

Abstract

Importance: Clear guidelines on the health effects of dairy food are important given the high prevalence of obesity, cardiovascular disease and diabetes, and increasing global consumption of dairy food.

Objective: To evaluate the effects of increased dairy food on cardio metabolic risk factors.

Data Sources: Searches were performed until April 2013 using MEDLINE, Science Direct, Google,Embase, the Cochrane Central Register of Controlled Trials, reference lists of articles, and proceedings of major meetings.

Study Selection: Randomized controlled studies with healthy adults randomized to increased dairy food for more than one month without additional interventions.

Data Extraction and Synthesis: A standard list was used to extract descriptive, methodological and key variables from all eligible studies. If data was not included in the published report corresponding authors were contacted.

Results: 20 studies with 1677 participants with a median duration of dietary change of 26 (IQR 10-39) weeks and mean increase in dairy food intake of 3.6 (SD 0.92) serves/day were included.

There was an increase in weight with low (+0.82, 0.35 to 1.28 kg, p<0.001) and whole fat dairy food (+0.41, 0.04 to 0.79kg, p=0.03), but no significant change in waist circumference (-0.07 , -1.24 to 1.10 cm) ; HOMA –IR (-0.94 , -1.93 to 0.04 units); fasting glucose (+1.32 , 0.19 to 2.45 mg/dl) ; LDL-c (1.85 ,-2.89 to 6.60 mg/dl); HDL-c (-0.19 , -2.10 to 1.71 mg/dl); systolic BP (-0.4, -1.6 to 0.8 mmHg); diastolic BP (-0.4 , -1.7 to 0.8 mmHg) or CRP (-1.07 , -2.54 to 0.39 mg/L). Changes in other cardio-metabolic risk factors were similar for low and whole fat dairy interventions.

Limitations: Most clinical trials were small and of modest quality. .

Conclusion: Increasing whole fat and low fat dairy food consumption increases weight but has minor effects on other cardio-metabolic risk factors.

Trial Registration ACTRN: Australian New Zealand Clinical Trials Registry ACTRN12613000401752, http://www.anzctr.org.au

Ethics Approval Number: NTX/10/11/115

Citation: Benatar JR, Sidhu K, Stewart RAH (2013) Effects of High and Low Fat Dairy Food on Cardio-Metabolic Risk Factors: A Meta-Analysis of Randomized Studies. PLoS ONE 8(10): e76480. doi:10.1371/journal.pone.0076480

Editor: Yu-Kang Tu, National Taiwan University, Taiwan

Received July 20, 2013; **Accepted** August 23, 2013; **Published** October 11, 2013

Copyright: © 2013 Benatar et al. This is an open-access article distributed under the terms of the Creative Commons Attribution License, which permits unrestricted use, distribution, and reproduction in any medium, provided the original author and source are credited.

Funding: The authors have no support or funding to report.

Competing interests: The authors have declared that no competing interests exist.

* E-mail: Jocelyne.Benatar@adhb.govt.nz

Introduction

Clear guidelines on the health effects of dairy food are important given the high and increasing prevalence of obesity[1], cardiovascular disease [2] and diabetes[3] in most countries, and the increasing global consumption of dairy food[4]. Many current dietary guidelines promote low fat dairy as a healthy food[5,6]. This advice is supported by observational studies which report that increased dairy consumption is associated with lower blood pressure[7-11], weight reduction [12], improved insulin sensitivity[7,11,13,14], less inflammation [15,16] and a lower ratio of total to HDL cholesterol[17]. A modest inverse association between dairy consumption and cardiovascular disease has also been reported[18-20].

In contrast, whole fat dairy is not recommended in most food guidelines [21-23] because of the concern that saturated fat in dairy food may have an adverse effect on serum lipids which

could increase the risk of cardiovascular disease. Despite these guidelines the effects of high fat dairy food on the risk of obesity, diabetes and cardiovascular disease are uncertain. A recent meta-analysis found no association of dietary saturated fat intake and the risk of cardiovascular disease[24]. Whole fat dairy foods contain many fatty acids, which may have favorable as well as unfavorable effects on lipids and other cardio-metabolic risk factors[25]. Also, effects of reducing saturated fat from one food are determined by other dietary changes, including carbohydrates, and mono-unsaturated and poly-unsaturated fatty acids[17].

The effects of a high dairy food diet on diabetes and cardiovascular disease have not been evaluated in randomized clinical outcome trials. The large long term randomized dietary intervention studies which evaluated the 'Dietary Approaches to Stop Hypertension' (DASH) [26] and 'Mediterranean' [27] diets on clinical outcomes, while including increased low fat dairy food in the intervention, do not allow an evaluation of the independent effects of changes in dairy food intake. Health effects of whole and low fat dairy food would be more reliably evaluated in clinical trials than in observational studies, and by assessing a number, rather than just one cardio-metabolic risk factor. We therefore undertook a meta-analysis of randomized clinical studies that evaluated effects of changing whole and low fat dairy food intake in healthy adults on a broad range of cardio-metabolic risk factors including weight, insulin resistance, lipids, blood pressure and c- reactive protein.

Methods

We followed the PRISMA (http://www.prisma-statement.Org) guidelines throughout the design, implementation, analysis, and reporting of this meta-analysis. A protocol for the study was designed and is available as File S2. The study was registered with the Australian New Zealand Clinical Trials Registry, with trial registration number ACTRN12613000401752.

Search Strategy

We searched for all trials that randomized adults to increased dairy for at least one month without additional interventions (e.g. caloric restriction, multiple dietary interventions), had an appropriate control group, and sufficient data to calculate estimates of effect with standard deviations on at least one of the following: weight, waist circumference, blood pressure, HDL and LDL cholesterol, fasting glucose, insulin resistance and C-reactive protein. Studies were excluded if they were observational or otherwise non-randomized; were commentaries, reviews, or duplicate publications from the same study. We restricted to studies of healthy adults who did not have diabetes, hypertension or vascular disease. Both feeding and dietary advice trials and studies with a crossover or parallel group study design were included.

Searches were performed of literature published through March 2013 using Medline, Science Direct, Google, Embase, the Cochrane Central Register of Controlled Trials, reference lists of articles, and proceedings of major meetings for relevant literature. The search terms were 'dairy' and each of the following; 'cardio metabolic risk', 'weight', 'waist circumference', 'glucose', 'insulin', 'insulin resistance', 'inflammation', 'inflammatory markers', 'blood pressure', 'cholesterol' and 'lipids'.

Assessment of study eligibility and data extraction

One reviewer screened all abstracts and titles and, upon retrieval of candidate studies, two team members (JB, KS) reviewed the full text to determine eligibility. If the study was eligible, data were abstracted by JB. Through an iterative process, a standard list was used to extract descriptive, methodological and key variables from all eligible studies. Data extracted included years the study was performed and reported, the primary aim of the study, population characteristics, funding source, control and intervention diets, duration of follow-up, estimates of effect and standard deviations. If data was not included in the published report corresponding authors were contacted [28,29]. The quality of each study was rated using the Jadad score[30]. Questions arising during data abstraction were resolved by discussion with all team members.

Definitions

Dairy food with less 1% fat, such as trim or low fat milk was categorized as a low fat dairy food. Dairy food that included full fat milk (3-4% fat), cheese, butter, cream and ice cream, was categorized as whole fat dairy food..

The method used to quantify insulin resistance was the homeostatic model assessment- Insulin resistance (HOMA-IR) [31]. This estimates steady state insulin sensitivity as units. The equation that is used is HOMA-IR = glucose (mmol/L) x insulin (munits/L) ÷22.5.

Statistical analysis

Each cardio-metabolic risk factor when on a higher and lower dairy diet was compared between cases and controls from the same study. For those studies with 3 treatment groups, comparison was made between the control and the high dairy food group. Effects were measured at least 4 weeks after randomization, with the final results used for studies with more than one measurement during follow up. A negative effect size means that dairy has a favorable effect on the cardio metabolic risk factor. Because increase in HDL-cholesterol is considered beneficial, positive and negative exponents were switched to maintain consistency in presentation. In one study the standard deviation was not reported [32] but calculated from the 95% confidence intervals.

For each cardio-metabolic risk factor the weighted mean change from baseline to follow up was calculated across all included studies within each randomized group. The inverse-variance method, whereby study differences are weighted according to the reciprocal of their variance, was used to pool all standardized mean differences to yield an overall effect size with corresponding 95% confidence intervals.

Each meta-analysis was assessed for heterogeneity by a Chi square test and I^2 statistic. A fixed effects model was used when heterogeneity was not present (I2=0) and a random effects model was used when statistical heterogeneity ($I^2 \geq 1\%$)

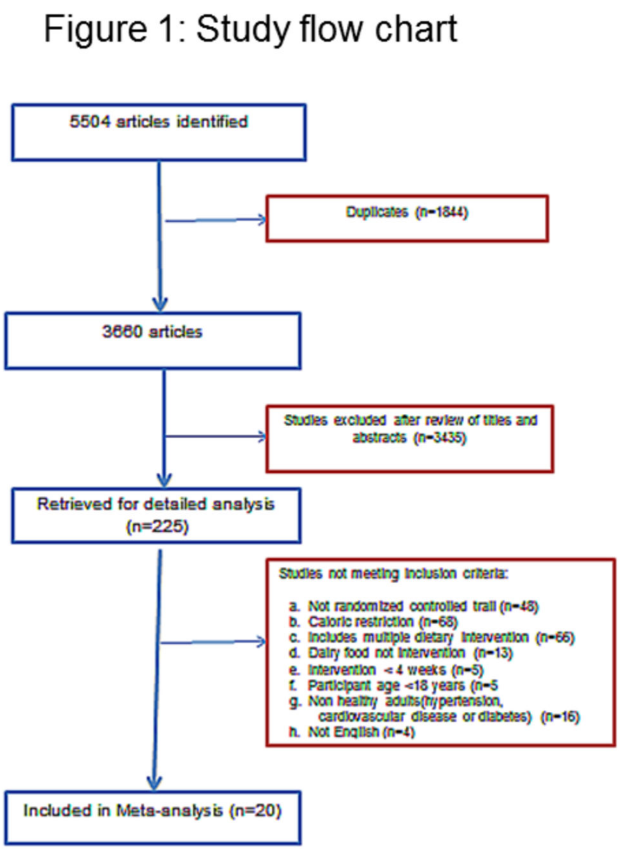

Figure 1. Study flow chart.

doi: 10.1371/journal.pone.0076480.g001

was present. A p-value of <0.05 was considered statistically significant. Studies are presented in Forrest plots in order of statistical power. Stratified analyses was studied by low fat and whole fat dairy, duration of dietary intervention (less than or greater than 6 months), body weight of study participants, and industry or public source of funding. Studies where the intervention was skim, trim or <1% dairy food are low fat dairy studies. Sensitivity analyses were also conducted to evaluate the impact of selected studies on overall pooled estimates and heterogeneity. The Statistical analyses were performed using RevMan software version 5·2 (The Nordic Cochrane Centre, The Cochrane Collaboration, Copenhagen).

Results

Search Results

The literature search yielded 5504 citations (2495 on Pub Med and 2849 on Science Direct,160 on Google), of which included 1844 duplicates. After title and abstract screening, the full text of 225 articles were evaluated, with 205 excluded either because the intervention included caloric restriction or had multiple dietary changes, or the study was in a population with disease (Figure 1). Twenty studies were included in the meta-analysis.

Characteristics of studies

Characteristics of the 20 included trials which included 1677 participants are summarized in Table 1. The average age was 51 (SD 16) years and 78% of participants were female. The median duration of follow up was 26 (IQR 10-39) weeks. The average difference in dairy intake between groups was 3.12 (SD 0.62) standard serving sizes /day. Within studies there was no imbalance between randomized groups. One crossover study [28] had a higher dropout rate for subjects initially randomized to low compared to high dairy intake (49% vs. 22%).

Sixteen studies had a parallel group design and 4 were cross-over studies. In 10 studies increased dairy food included whole fat dairy, while 10 only low fat dairy food was advised. Fifteen of the studies were at least partly funded by the dairy or food industry.

Change in risk factors on a high and a lower dairy diet in all studies combined are displayed in figures 2-10. Results stratified by duration of intervention, participant body weight and by funding source are displayed in table 2.

Effects on body weight

Eighteen [10,28,29,32-46] studies reported effects on weight in 1629 individuals (Figure 2). The mean body mass index (BMI) at baseline was 25.6(SD 6.2) kg/m^2 and weight 77.7(SD

Table 1. Baseline characteristics of studies included in this meta-analysis.

Trial Year published	Country	Population	Mean BMI (kg/M 2)	Funding source	Number of subjects (% female)	Mean Age years (SD)	Design	Length of intervention (Weeks)	Primary outcome	Low dairy	High Dairy (+ serves / day)	Jadad Score
Alonso[32] 2009	Spain	Normotensive college students	23.6	Public	45 (60%)	19·9 (1·5)	Crossover	8	Blood pressure and weight	1·5 serves/day	3·5 whole-fat	2
Baran[46] 1990	America	Healthy volunteers	22.8	Industry	37 (100%)	36.5 (3.4)	Parallel	156	Bone density	Usual diet	2.5 whole-fat or low-fat dairy	2
Barr[29] 2000	North America	Healthy volunteers	25.8	Industry	198 (64%)	65·2 (6·7)	Parallel	12	Weight, blood pressure, lipids	Usual diet	3 low-fat dairy	3
Benatar[67] 2013	New Zealand	Healthy volunteers	24.6	Public	120 (75%)	46·3 (12·0)	Parallel	4	Weight, blood pressure	Usual diet	3·5 whole-fat or low-fat dairy	3
Chee[36] 2003	Malaysia	Postmenopausal Chinese woman	23.8	Industry	173 (100)	59·0 (3·2)	Parallel	104	Bone loss	Usual diet	2 low-fat dairy	3
Crichton[68] 2012	Australia	Overweight and obese volunteers	31.5	Public	36 (83)	47·3 (15·1)	Crossover	26	Weight	≤1 serves/day	4 low-fat dairy	2
Eagan[37] 2006	North America	Normal weight young women	22.5	Industry	37 (100)	20·0 (2·0)	Parallel	26	Fat mass	Usual diet	3 whole-fat or low-fat dairy	2
Gardner[47] 2007	North America	Healthy volunteers	26.0	Industry	28 (79)	52(9)	Crossover	4	Lipids	Usual diet	2·5 low-fat dairy	3
Ghadirian [35] 1995	Canada	Postmenopausal nuns	23.0	Industry	158(100%)	79 (9.5)	Parallel	4	Uric acid	0	3.6 whole-fat or low-fat dairy	2
Gunther [38] 2005	North America	Healthy woman	22.2	Industry	99 (100)	20·0 (2·1)	Parallel	52	Weight	Usual diet	3 low-fat dairy	2
Kukuljan [34] 2009	Australia	Older men (>50years)	27.6	Public	89(0%)	60.9 (7.5)	Parallel	52	Bone Density	Usual diet	1.7 low-fat dairy	2
Lau[39] 2001	China	Postmenopausal women	22.2	Industry	185 (100)	57·0 (1·8)	Parallel	104	Bone loss	Usual diet	2 low-fat dairy	3
Manios[33] 2009	Greece	Postmenopausal women	30.4	Industry	62(100)	61.2 (4.9)	Parallel	52	Weight	Usual diet	3 low-fat dairy	2
Palacios[40] 2011	Puerto Rica	Obese adults	38.5	Public	16 (81)	37·0 (2·2)	Parallel	21	Weight, lipids	Usual diet	4 whole-fat or low-fat dairy	1
Stancliffe[41] 2011	North America	Overweight and obese with metabolic syndrome	30.7	Industry	40 (53)	37·0 (9·9)	Parallel	12	Inflammatory markers	0·5 serves /day	3·5 whole-fat or low-fat dairy	2

Table 1 (continued).

Trial Year published	Country	Population	Mean BMI (kg/M 2)	Funding source	Number of subjects (% female)	Mean Age years (SD)	Design	Length of intervention (Weeks)	Primary outcome	Low dairy	High Dairy (+ serves / day)	Jadad Score
Tardy[42] 2009	France	Healthy women with abdominal obesity	32.6	Public	39 (100)	36·4 (7·7)	Parallel	4	HOMA	Usual diet + vegetable oils	3 whole-fat	2
Van Meilj[49] 2010	Holland	Overweight adults	32.0	Industry	35 (71)	49·5(13·2)	Crossover	8	Blood pressure, Lipids, inflammatory marker, glucose	Usual diet	3 low-fat dairy	2
Wennesberg[43] 2009	Scandinavia	Metabolic syndrome	30.0	Industry and Public	105 (67)	51·2 (8·0)	Parallel	26	Waist circumference	Usual diet	3-5 whole-fat or low-fat dairy	2
Zemell[48] 2010	North America	Obese and overweight adults	30.0	Industry	20 (30)	31·0 (10·3)	Crossover	4	Inflammatory markers	0	3 low-fat dairy	3
Zemmel (phase 1)[44] 2005	North America	Obese adults	34.9	Industry	34 (59)	41·7 (2·8)	Parallel	26	Weight	Usual diet	3 whole-fat or low-fat dairy	1

doi: 10.1371/journal.pone.0076480.t001

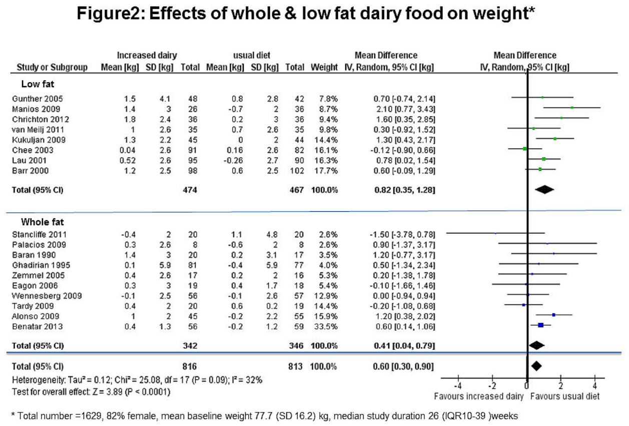

Figure 2. Effects of whole & low fat dairy food on weight.

doi: 10.1371/journal.pone.0076480.g002

16.2) kg. Increased dairy intake was associated with a modest weight gain (+0.60, 95% confidence interval 0.30 to 0.90kg, p<0.0001). In six studies[28,33,41-43,45] in 440 individuals waist circumference did not change significantly(-0.07 , -1.24 to 0. 1.10cm) (Figure 3).

Weight gain was observed both in studies which increased low fat (+0.82, 0.35 to 1.28 kg, p<0.001) and whole fat dairy food (+0.41, 0.04 to 0.79kg, p=0.03). Modest weight gain was also observed in 10 studies (n= 692) which included overweight and obese subjects (+0.60, 0.01 to 1.19kg, p=0.03) and in 8 studies (n= 937) of normal weight participants (+0.59, 0.31 to 0.87kg, p=0.02).

Effects on insulin resistance

In 8 studies [10,28,29,35,41,42,45,47], there was no significant change in fasting glucose on higher compared to lower dairy diet (Figure 4). Four studies [41-43,45] assessed effects on HOMA-IR in 270 subjects (Figure 4). One [42] did

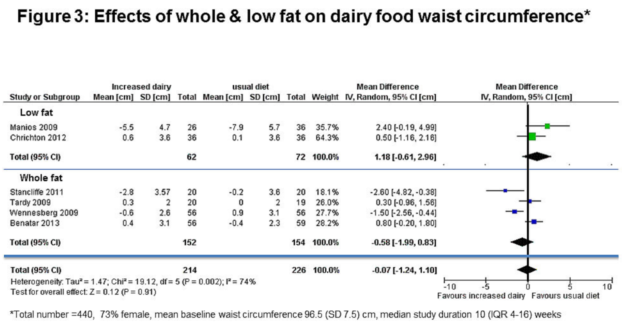

Figure 3. Effects of whole & low fat dairy food on waist circumference.

doi: 10.1371/journal.pone.0076480.g003

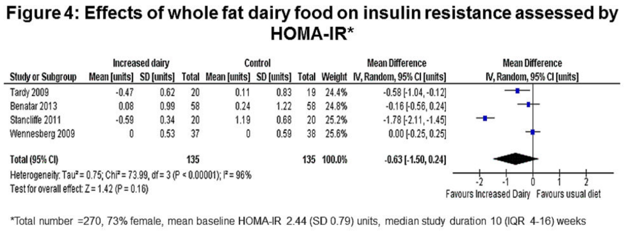

Figure 4. Effects of whole & low fat dairy food on Insulin resistance as measured by HOMA-insulin resistance.

doi: 10.1371/journal.pone.0076480.g004

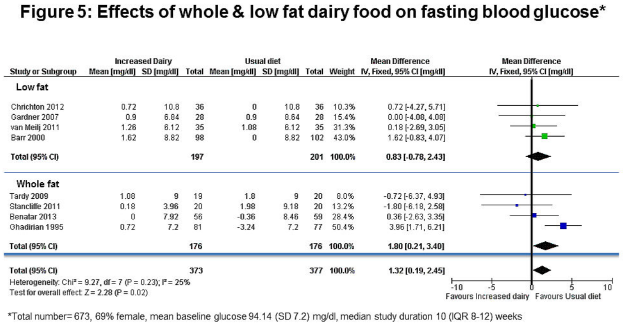

Figure 5. Effects of whole & low fat dairy food on fasting glucose levels.

doi: 10.1371/journal.pone.0076480.g005

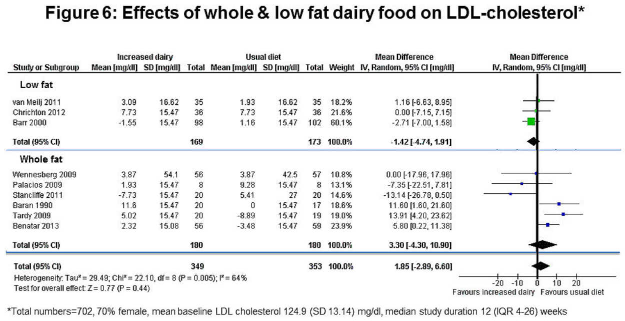

Figure 6. Effects of whole & low fat dairy food on LDL-cholesterol.

doi: 10.1371/journal.pone.0076480.g006

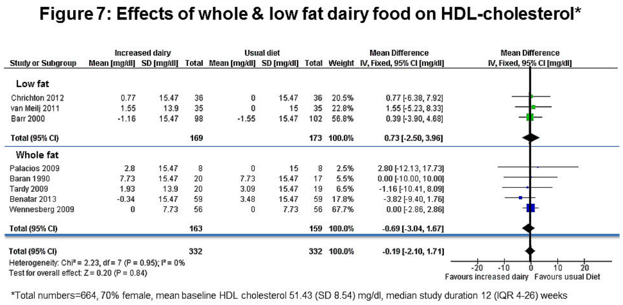

Figure 7. Effects of whole & low fat dairy food on HDL-cholesterol.

doi: 10.1371/journal.pone.0076480.g007

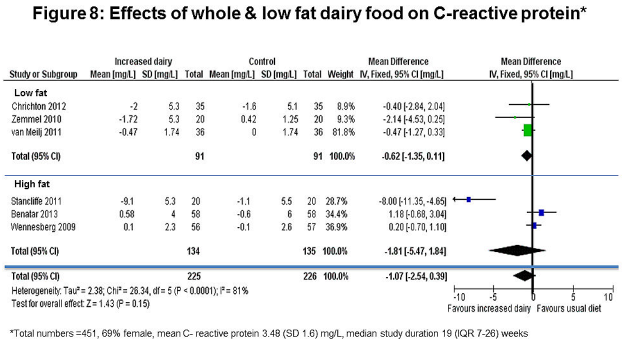

Figure 8. Effects of whole & low fat dairy food on C-reactive protein.

doi: 10.1371/journal.pone.0076480.g008

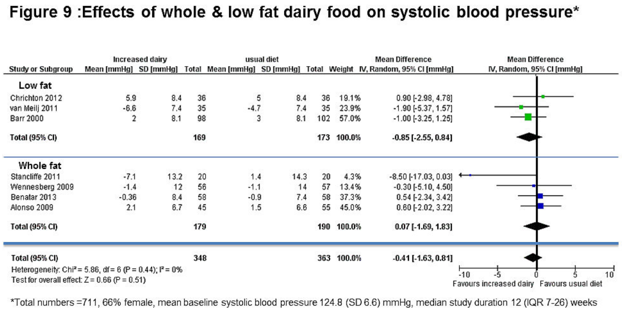

Figure 9. Effects of whole & low fat dairy food on systolic blood pressure.

doi: 10.1371/journal.pone.0076480.g009

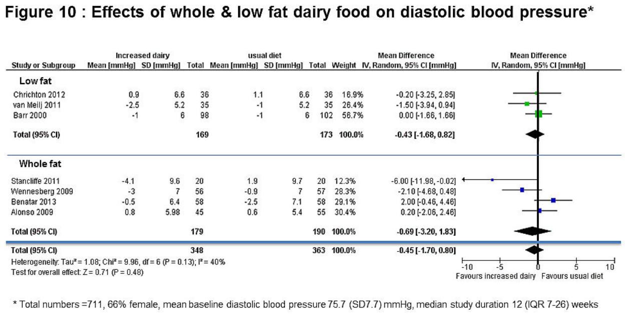

Figure 10. Effects of whole & low fat dairy food on diastolic blood pressure.

doi: 10.1371/journal.pone.0076480.g010

Table 2. Studies stratified by study duration, study population normal weight or overweight and source of funding on cardio-metabolic risk factors.

Metabolic risk factors	Diet Change <6 months duration	Diet change ≥ 6 months duration	Normal weight (BMI <25 kg/m^2)	Overweight or obese (BMI > 25kg/m^2)	Industry funded studies	Public funded studies
Total number of studies	10	10	8	12	14	6
Total number of subjects	738	982	854	823	1246	431
**Weight (kg)**	+0.51* (0.14 to 0.88)	+0.74* (0.24 to 1.20)	+0.59* (0.29 to 0.89)	+0.61* (0.03 to1.24)	+0.44* (0.12 to 0.76)	+0.82* (0.32 to 1.33)
N	738	891	895	534	1198	431
**Waist circumference (cm)**	+0.24 (-1.22 to 0.70)	+0.22 (-1.91 to 2.35)	+0.80 ([-0.20 to 1.80) n=115	-0.29 (-1.67 to 1.10)	-0.73 (-3.11 to 1.65)	+0.59 (-0.12 to 1.29)
N	194	246	115	325	214	226
**HOMA- IR (units)**	-0.85 (-1.88 to 0.19)	0.00 (-0.25 to 0.25)	-0.16 (-0.56 to 0.24)	-0.79 (-1.94 to 0.37)	-1.59 (-4.77 to 1.59)	-0.40* (-1.01 to 0.21)
N	195	75	116	154	115	155
**Fasting Glucose (mg/dl)**	+0.36 (-0.90 to 1.80)	+0.72 (-4.32 to 5.76)	+0.36 (-2.70 to 3.42)	+0.36 (-1.08 to 1.80)	+0.54 (-1.08 to 2.16)	+0.18 (-2.16 to 2.52)
N	601	72	196	477	477	226
**LDL-cholesterol (mg/dl)**	+0.87 (-5.03 to 4.25)	+4.25 (-4.25 to 12.37)	+5.8* (0.39 to 11.2)	0.00 (-4.25 to 4.25)	-0.00 (-6.57 to 6.57)	+2.70 (-1.54 to 6.96)
N	480	222	115	587	460	242
**HDL-cholesterol (mg/dl)**	-1.16 (-4.25 to 1.55)	+0.00 (-2.32 to 2.70)	-3.09 (-7.73 to 1.93)	-0.00 (-1.93 to 1.93)	+ 1.16 (-6.12 to 8.50)	-6.57 (-16.24 to 3.48)
N	443	221	155	509	419	245
**C- reactive protein (mg/L)**	-1.97 (-4.61 to 0.67)	+0.13 (-0.72 to 0.98	+1.18 ([-0.68 to 3.04)	-1.56 (-3.22 to 0.10)	-1.89 (-3.84 to 0.07)	0.60 (-0.88 to 2.08)
N	268	183	116	335	265	186
**Systolic blood pressure (mmHg)**	-0.57 (-1.91 to 0.76)	+0.43 (-2.59 to 3.45)	+0.57 (-1.37 to 2.51)	-1.05 (-2.62 to 0.52)	-1.44 (-3.15 to 0.28)	+0.64 (-1.10 to 2.37)
N	526	185	216	495	423	288
**Diastolic blood pressure (mmHg)**	-0.21 (-1.78 to 1.36)	-1.31 (-3.28 to 0.66)	+1.03 (-0.73 to 2.79)	-1.08 (-2.41 to 0.26	-1.37 (-3.05 to 0.30)	+0.74 (-0.72 to 2.20)
N	526	185	216	495	423	423

*. P <0.05 n = number of participants

doi: 10.1371/journal.pone.0076480.t002

not report standard deviation so a weighted mean standard deviation from the other studies was used. HOMA-IR was recalculated in one study which used incorrect units [43]. For all studies combined HOMA-IR was slightly improved on the high dairy diet (-0.94, -1.93 to 0.04 units, p=0.06). However there was heterogeneity between studies (I^2 =92%), accounted for by the two smallest studies [41,42] which reported reduced insulin resistance on the high dairy diet (-1.37, -1.64 to -1.10 units). HOMA-IR was similar on high and low dairy diets in the two larger studies[43,45] (-0.05, -0.26 to 0.17 units). HOMA-IR did not change significantly for studies stratified by body weight, duration of intervention or high versus low fat dairy (table 2).

Effects on LDL and HDL cholesterol

Nine studies [10,28,29,40-43,45,46] assessed effects on LDL- and HDL-cholesterol in 702 individuals. For all studies combined there was no significant change in either LDL or HDL-cholesterol after increasing dairy food (figure 8 and 9). Effects of HDL-cholesterol were consistent (I^2=0%) across studies, but there was heterogeneity for LDL-cholesterol (I^2=49%). There was no change in LDL cholesterol when whole fat dairy (+3.30, -4.30 to 10.90mg/dl]) or low fat dairy (-1.42 ,-4.74, to 1.91mg/dl) food was increased. Results were similar for shorter and longer periods of dietary intervention and for studies which included normal and overweight or obese participants.

Effects on C-reactive protein

Six studies[28,41,43,45,48,49] assessed effects on C-reactive protein in 400 individuals (Figure 10). For all studies combined there was no significant change in C-reactive protein on a high dairy diet. However two smaller studies [41,48] reported significant reductions in C-reactive protein with increased dairy intake (-1.10, -2.27 to 0.06mg/L). There was no evidence for effects on C-reactive protein when studies were

stratified by duration of dietary intervention, high and low fat dairy, or normal or overweight subjects (Table 2).

Effects on blood pressure

Seven studies [10,28,29,32,41,43,45,49] assessed effects on blood pressure in 711 participants (figure 6 and 7). For all studies there was no significant change in either systolic blood pressure or diastolic blood pressure. There was also no evidence for effects on blood pressure when studies were stratified by duration of dietary intervention, high and low fat dairy or normal or overweight subjects.

Evaluation of heterogeneity and sensitivity analysis

Industry sponsored studies were more likely to report favorable effects on risk factors than non-industry sponsor studies (table 2). . Results were also similar when analysis was repeated excluding the 4 cross-over studies. Funnel plots identified that the study by Stancliffe [41] reported decreases in LDL cholesterol, HOMA-IR, C-reactive protein and waist circumference on the increased dairy diet beyond the 95% confidence range for all studies combined. This study also reported the greatest decrease in blood pressure and weight of all studies. Excluding this study in a sensitivity analysis substantially decreased heterogeneity, but overall effects were similar, and there was no other consistent difference between smaller and larger studies. The study by Manios[33] was the only study that fell outside the 95% confidence interval for weight, though excluding the study had no overall effect on results. This study had an imbalance in randomization with fewer people randomized to high dairy food (n=30) compared to the control group (n=40). The study by Ghadirian[35] was outside the 95% confidence interval for fasting plasma glucose. . In this study, the control group had a statistically significant reduction in fasting plasma glucose, with little change for the high dairy group.. Analysis repeated excluding this study showed no overall effect (+0.02,-0.05 to 0.10mmol/L, p=0.53) and a reduction in heterogeneity (25→0%).

On the basis of a funnel plot and Begg's test, no significant publication bias was shown in the meta-analysis of body weight, waist circumference, insulin resistance, blood pressure, lipids and C-reactive protein.

Discussion

This systematic analysis of randomized dietary intervention trials suggests that a moderate increase in dairy food consumption has no or small effects on the major cardiovascular and metabolic risk factors [50]. This conclusion contrasts with results from several large epidemiological studies, which concluded that dairy food may have favorable effects on insulin resistance and decrease the risk of type 2 diabetes[7,51]. However, in these observational studies[32,52-54] dairy food intake was associated with an overall healthier eating pattern, healthier lifestyle, higher socio economic status and educational attainment, which are each associated with more favorable cardio metabolic profiles[55]. Evaluating effects of dairy in randomized intervention trials is likely to be more reliable than from observational studies, where associations may not be causal.

Several observational studies have suggested that dairy food may facilitate weight loss, particularly in obese and overweight individuals [12]. Also in randomized trials where the intervention included both increased dairy food and caloric restriction, weight loss has been reported [56]. However, in the current meta-analysis, which included studies which gave no advice on calorie restriction, increasing dairy food resulted in a modest weight gain. Results were similar in studies which included overweight and obese participants. Whilst no direct comparison is possible, mean weight gain on low fat dairy food is double that of whole fat dairy food. This is counterintuitive but is in keeping with a recent viewpoint in JAMA pediatrics[57] which suggest that trim milk is associated with increased weight in children. It is likely the weight gain was the result of increased total calories in studies which encouraged greater dairy intake without other changes in diet. It is uncertain whether weight gain also occurs when dairy food is taken as part of, rather than in addition to a balanced diet.

Several diabetes guidelines [5,58,59] recommend regular intake of low fat dairy because of its' low glycemic index[60]. In observational studies[51,61] persons in the highest quartile of dairy consumption have less insulin resistance, and this association is strongest in those who are overweight or obese. In this analysis insulin sensitivity improved in two small studies[41,42] with no effect in the larger trials[43,45]. In stratified analyses there was no effect in overweight and obese participants, or with whole or low fat dairy interventions. Based on these observations, it is uncertain whether increasing dairy food improves insulin sensitivity, and further well designed studies are needed to resolve this question. A recent review by the American Diabetes Association[62] concluded that 'none of the components of dairy appear to have an effect on glycemic control or cardiovascular disease risk reduction', consistent with this analysis.

Many food guidelines encourage low fat dairy food, but advise avoiding whole fat dairy [22,63]. In the current analysis LDL cholesterol did not change significantly when whole fat dairy consumption was increased. Whilst the risk of cardiovascular disease is reduced when saturated fats are replaced by unsaturated fats [64], the reasons for this may be multifactorial. This study suggests that effects on LDL-cholesterol may not the primary reason.

Dairy intake was associated with lower blood pressure in observational studies [8,13] and in the large randomized DASH study [26]. In the DASH study the intervention included increased low fat dairy food, reduced total and saturated fat, and increased fruit and vegetables. In a secondary analysis it was estimated that low fat dairy could account for about half of the observed 5.5mmHg decrease in systolic blood pressure. However it is not possible to reliably estimate the effects of each dietary component when the intervention includes multiple dietary changes. For this reason the DASH study and studies of the Mediterranean diet [27] were not included in this meta-analysis. In this meta-analysis, the confidence intervals exclude significant effects (>1.6mmHg) of increasing dairy food on systolic and diastolic blood pressure.

Limitations of meta-analysis

The majority of subjects included in the meta-analysis were women, but there is currently no evidence for different effects of diet by gender. The diverse population and age range of subjects included makes the results relevant to improving lifestyle risk factors for diabetes and cardiovascular disease in the general population. Studying healthy populations also avoids possible treatment and disease effects on the outcomes of interest. Further research is needed to confirm similar neutral effects of dairy in patients with established diabetes and cardiovascular disease.

It is possible the duration of the dietary intervention was not long enough to identify effects on risk factors, but stratified analyses suggest similar results for longer and shorter periods of dietary intervention. The increase of 3.6 servings each day is a substantial dietary change, and previous studies suggest dietary interventions influence risk factors within one month[26,65].

Most studies included were relatively small. It is difficult to blind diet studies and the level of compliance with the dietary interventions was often uncertain. Several studies reported significant adverse or favorable effects of increasing dairy food on one or more risk factors, but sensitivity analyses suggested these studies had only a small effect on overall estimates. It is also possible that smaller studies which found no effects have not been published. Three quarters of the studies were funded by the dairy or food industry, and results were more favorable for industry compared to non-industry funded studies. This meta-analysis stratified studies by ‘low fat’ and ‘whole fat’ interventions, but a direct comparison of these studies may not be reliable, and no trials which directly compare ‘low’ with ‘whole’ fat dairy diets have been reported. Because studies were small, and some may be unreliable, the analysis can not exclude a small increase in LDL cholesterol with increase in whole fat dairy consumption. Studies are also needed to evaluate effects of other components of dairy food.

To provide a better estimate of health effects the meta-analysis evaluated associations with multiple rather than just one or two risk factors. However dairy food could influence the risk of cardiovascular disease or diabetes by pathways other than the risk factors measured[66]. The influence of dairy food on clinical outcomes rather than risk factors is most important for dietary guidelines. However, currently, no completed randomized trials allow independent assessment of the effects of changing dairy food on diabetic complications or cardiovascular events.

Conclusion

Increase in both whole and low fat dairy food, without other dietary interventions, is associated with a modest weight gain, with no or minor effects on other cardio-metabolic risk factors. These observations suggest that for most healthy individuals it is reasonable to include both low and whole fat dairy food as part of a healthy diet.

Supporting Information

Checklist S1. Prisma checklist.
(DOC)

File S1. Prisma flow chart.
(PDF)

File S2. Protocol.
(DOCX)

Author Contributions

Conceived and designed the experiments: JB RS. Performed the experiments: JB KS. Analyzed the data: JB RS KS. Wrote the manuscript: JB RS.

References

1. Swinburn BA, Sacks G, Hall KD, McPherson K, Finegood DT et al. (2011) The global obesity pandemic: shaped by global drivers and local environments. Lancet 378: 804-814. doi:10.1016/S0140-6736(11)60813-1. PubMed: 21872749.
2. Organisation WH (2012) The Atlas of Heart Disease and Stroke. In: Organisation WH, editor.
3. Federation ID (2011) IDF Diabetes Atlas. 5th ed. Brussels, Belgium: International Diabetes Federation.
4. Development TOfEC-oa (2011); OECD FAO Agricultural Outlook 2011-2020.
5. American Diabetes Association, Bantle JP, Wylie-Rosett J, Albright AL, Apovian CM, Clark NG, Franz MJ, Hoogwerf BJ, Lichtenstein AH, Mayer-Davis E, Mooradian AD, Wheeler MLAmerican Diabetes A, Bantle JP, Wylie-Rosett J, Albright AL, Apovian CM, et al (2008) Nutrition recommendations and interventions for diabetes: a position statement of the American Diabetes Association. Diabetes Care 31 Suppl 1: S61-S78. doi:10.2337/dc08-0677. PubMed: 18165339.
6. Agriculture UDo (2012). US ChooseMyPlate.
7. Pereira MA, Jacobs DR Jr., Van Horn L, Slattery ML, Kartashov AI et al. (2002) Dairy consumption, obesity, and the insulin resistance syndrome in young adults: the CARDIA Study. JAMA 287: 2081-2089. doi:10.1001/jama.287.16.2081. PubMed: 11966382.
8. Toledo E, Delgado-Rodríguez M, Estruch R, Salas-Salvadó J, Corella D et al. (2009) Low-fat dairy products and blood pressure: follow-up of 2290 older persons at high cardiovascular risk participating in the PREDIMED study. Br J Nutr 101: 59-67. doi:10.1017/S0007114508981496. PubMed: 18492300.
9. Engberink MF, Geleijnse JM, de Jong N, Smit HA, Kok FJ et al. (2009) Dairy intake, blood pressure, and incident hypertension in a general Dutch population. J Nutr 139: 582-587. doi:10.3945/jn.108.093088. PubMed: 19158223.
10. van Meijl LE, Mensink RP (2011) Low-fat dairy consumption reduces systolic blood pressure, but does not improve other metabolic risk parameters in overweight and obese subjects. Nutr Metab Cardiovasc Dis 21: 355-361. doi:10.1016/j.numecd.2009.10.008. PubMed: 20153619.
11. Lutsey PL, Steffen LM, Stevens J (2008) Dietary intake and the development of the metabolic syndrome: the Atherosclerosis Risk in Communities study. Circulation 117: 754-761. doi:10.1161/CIRCULATIONAHA.107.716159. PubMed: 18212291.
12. Abargouei AS, Janghorbani M, Salehi-Marzijarani M, Esmaillzadeh A (2012) Effect of dairy consumption on weight and body composition in adults: a systematic review and meta-analysis of randomized controlled clinical trials. Int J Obes (Lond), 36: 1485–93. PubMed: 22249225.

13. Liu S, Song Y, Ford ES, Manson JE, Buring JE et al. (2005) Dietary calcium, vitamin D, and the prevalence of metabolic syndrome in middle-aged and older U.S. women. Diabetes Care 28: 2926-2932. doi: 10.2337/diacare.28.12.2926. PubMed: 16306556.
14. Snijder MB, van der Heijden AA, van Dam RM, Stehouwer CD, Hiddink GJ et al. (2007) Is higher dairy consumption associated with lower body weight and fewer metabolic disturbances? The Hoorn Study. Am J Clin Nutr 85: 989-995. PubMed: 17413097.
15. Panagiotakos DB, Pitsavos CH, Zampelas AD, Chrysohoou CA, Stefanadis CI (2010) Dairy products consumption is associated with decreased levels of inflammatory markers related to cardiovascular disease in apparently healthy adults: the ATTICA study. J Am Coll Nutr 29: 357-364. doi:10.1080/07315724.2010.10719852. PubMed: 21041810.
16. Esmaillzadeh A, Azadbakht L (2010) Dairy consumption and circulating levels of inflammatory markers among Iranian women. Public Health Nutr 13: 1395-1402. doi:10.1017/S1368980009992126. PubMed: 20003635.
17. Mensink RP, Zock PL, Kester AD, Katan MB (2003) Effects of dietary fatty acids and carbohydrates on the ratio of serum total to HDL cholesterol and on serum lipids and apolipoproteins: a meta-analysis of 60 controlled trials. Am J Clin Nutr 77: 1146-1155. PubMed: 12716665.
18. Hu FB, Stampfer MJ, Manson JE, Ascherio A, Colditz GA et al. (1999) Dietary saturated fats and their food sources in relation to the risk of coronary heart disease in women. Am J Clin Nutr 70: 1001-1008. PubMed: 10584044.
19. Kelemen LE, Kushi LH, Jacobs DR Jr., Cerhan JR (2005) Associations of dietary protein with disease and mortality in a prospective study of postmenopausal women. Am J Epidemiol 161: 239-249. doi: 10.1093/aje/kwi038. PubMed: 15671256.
20. Elwood PC, Strain JJ, Robson PJ, Fehily AM, Hughes J et al. (2005) Milk consumption, stroke, and heart attack risk: evidence from the Caerphilly cohort of older men. J Epidemiol Community Health 59: 502-505. doi:10.1136/jech.2004.027904. PubMed: 15911647.
21. Guidelines ED European Dietary Guidelines
22. American Heart Association Nutrition; American Heart Association Nutrition Committee, Lichtenstein AH, Appel LJ, Brands M, Carnethon M, Daniels S, Franch HA, Franklin B, Kris-Etherton P, Harris WS, Howard B, Karanja N, Lefevre M, Rudel L, Sacks F, Van Horn L, Winston M, Wylie-Rosett JC, Lichtenstein AH, Appel LJ, Brands M, Carnethon M, et al (2006) Diet and lifestyle recommendations revision 2006: a scientific statement from the American Heart Association Nutrition Committee. Circulation 114: 82-96. doi:10.1161/CIRCULATIONAHA.106.617829. PubMed: 16785338.
23. Recommendations AD (2012). Aust Dietary Recommendations.
24. Siri-Tarino PW, Sun Q, Hu FB, Krauss RM (2010) Meta-analysis of prospective cohort studies evaluating the association of saturated fat with cardiovascular disease. Am J Clin Nutr 91: 535-546. doi:10.3945/ajcn.2009.27725. PubMed: 20071648.
25. Iggman D, [!(surname)!] (2011) Role of Different Dietary Saturated Fatty Acids for Cardiometabolic Risk. J Clin Lipidol 6: 209-223. doi: 10.2217/clp.11.7.
26. Appel LJ, Moore TJ, Obarzanek E, Vollmer WM, Svetkey LP et al. (1997) A clinical trial of the effects of dietary patterns on blood pressure. DASH Collaborative Research Group. N Engl J Med 336: 1117-1124. doi:10.1056/NEJM199704173361601. PubMed: 9099655.
27. Estruch R, Ros E, Salas-Salvado J, Covas MI et al. (2013) Primary Prevention of Cardiovascular Disease with a Mediterranean Diet. N Engl J Med.
28. Crichton GE, PR CH, Buckley JD, Coates AM, Murphy KJ (2012) Dairy consumption and cardiometabolic health: outcomes of a 12-month crossover trial. Nutr Metab (Lond) 9: 19. doi:10.1186/1743-7075-9-19. PubMed: 22433747.
29. Barr SI, McCarron DA, Heaney RP, Dawson-Hughes B, Berga SL et al. (2000) Effects of increased consumption of fluid milk on energy and nutrient intake, body weight, and cardiovascular risk factors in healthy older adults. J Am Diet Assoc 100: 810-817. doi:10.1016/S0002-8223(00)00236-4. PubMed: 10916520.
30. Jadad AR, Caroll D (1996) Assessing the quality of reports of randomized clinical trials: is blinding necessary? Control Clin Trials 17: 1-12. doi:10.1016/S0197-2456(96)90740-0. PubMed: 8721797.
31. Levy JC, Matthews DR, Hermans MP (1998) Correct homeostasis model assessment (HOMA) evaluation uses the computer program. Diabetes Care 21: 2191-2192. doi:10.2337/diacare.21.12.2191. PubMed: 9839117.
32. Alonso A, Zozaya C, Vázquez Z, Alfredo Martínez J, Martínez-González MA (2009) The effect of low-fat versus whole-fat dairy product intake on blood pressure and weight in young normotensive adults. J Hum Nutr Diet 22: 336-342. doi:10.1111/j.1365-277X.2009.00967.x. PubMed: 19486260.
33. Manios Y, Moschonis G, Koutsikas K, Papoutsou S, Petraki I et al. (2009) Changes in body composition following a dietary and lifestyle intervention trial: The postmenopausal health study. Maturitas 62: 58-65. doi:10.1016/j.maturitas.2008.11.005. PubMed: 19118956.
34. Kukuljan S, Nowson CA, Bass SL, Sanders K, Nicholson GC et al. (2009) Effects of a multi-component exercise program and calcium-vitamin-D3-fortified milk on bone mineral density in older men: a randomised controlled trial. Osteoporos Int 20: 1241-1251. doi:10.1007/s00198-008-0776-y. PubMed: 18958384.
35. Ghadirian P, Shatenstein B, Verdy M, Hamet P (1995) The influence of dairy products on plasma uric acid in women. Eur J Epidemiol 11: 275-281. doi:10.1007/BF01719431. PubMed: 7493659.
36. Chee WS, Suriah AR, Chan SP, Zaitun Y, Chan YM (2003) The effect of milk supplementation on bone mineral density in postmenopausal Chinese women in Malaysia. Osteoporos Int 14: 828-834. doi:10.1007/s00198-003-1448-6. PubMed: 12915959.
37. Eagan MS, Lyle RM, Gunther CW, Peacock M, Teegarden D (2006) Effect of 1-year dairy product intervention on fat mass in young women: 6-month follow-up. Obesity (Silver Spring) 14: 2242-2248. doi:10.1038/oby.2006.263. PubMed: 17189552.
38. Gunther CW, Legowski PA, Lyle RM, McCabe GP, Eagan MS et al. (2005) Dairy products do not lead to alterations in body weight or fat mass in young women in a 1-y intervention. Am J Clin Nutr 81: 751-756. PubMed: 15817848.
39. Lau EM, Woo J, Lam V, Hong A (2001) Milk supplementation of the diet of postmenopausal Chinese women on a low calcium intake retards bone loss. J Bone Miner Res 16: 1704-1709. doi:10.1359/jbmr.2001.16.9.1704. PubMed: 11547841.
40. Palacios C, Bertrán JJ, Ríos RE, Soltero S (2011) No effects of low and high consumption of dairy products and calcium supplements on body composition and serum lipids in Puerto Rican obese adults. Nutrition 27: 520-525. doi:10.1016/j.nut.2010.02.011. PubMed: 20579848.
41. Stancliffe RA, Thorpe T, Zemel MB (2011) Dairy attentuates oxidative and inflammatory stress in metabolic syndrome. Am J Clin Nutr 94: 422-430. doi:10.3945/ajcn.111.013342. PubMed: 21715516.
42. Tardy AL, Lambert-Porcheron S, Malpuech-Brugère C, Giraudet C, Rigaudière JP et al. (2009) Dairy and industrial sources of trans fat do not impair peripheral insulin sensitivity in overweight women. Am J Clin Nutr 90: 88-94. doi:10.3945/ajcn.2009.27515. PubMed: 19474135.
43. Wennersberg MH, Smedman A, Turpeinen AM, Retterstøl K, Tengblad S et al. (2009) Dairy products and metabolic effects in overweight men and women: results from a 6-mo intervention study. Am J Clin Nutr 90: 960-968. doi:10.3945/ajcn.2009.27664. PubMed: 19710195.
44. Zemel MB, Richards J, Milstead A, Campbell P (2005) Effects of calcium and dairy on body composition and weight loss in African-American adults. Obes Res 13: 1218-1225. doi:10.1038/oby.2005.144. PubMed: 16076991.
45. Benatar JR, Jones E, White HD, Stewart RAH (2013) A randomized trial evaluating the effects of change in dairy food consumption on cardio-metabolic risk factors. European. Prev Cardiol.
46. Baran D, Sorensen A, Grimes J, Lew R, Karellas A et al. (1990) Dietary modification with dairy products for preventing vertebral bone loss in premenopausal women: a three-year prospective study. J Clin Endocrinol Metab 70: 264-270. doi:10.1210/jcem-70-1-264. PubMed: 2294135.
47. Gardner CD, Messina M, Kiazand A, Morris JL, Franke AA (2007) Effect of two types of soy milk and dairy milk on plasma lipids in hypercholesterolemic adults: a randomized trial. J Am Coll Nutr 26: 669-677. doi:10.1080/07315724.2007.10719646. PubMed: 18187432.
48. Zemel MB, Sun X, Sobhani T, Wilson B (2010) Effects of dairy compared with soy on oxidative and inflammatory stress in overweight and obese subjects. Am J Clin Nutr 91: 16-22. doi:10.3945/ajcn.2009.28468. PubMed: 19889829.
49. van Meijl LE, Mensink RP (2010) Effects of low-fat dairy consumption on markers of low-grade systemic inflammation and endothelial function in overweight and obese subjects: an intervention study. Br J Nutr 104: 1523-1527. doi:10.1017/S0007114510002515. PubMed: 20579405.
50. Yusuf S, Hawken S, Ounpuu S, Dans T, Avezum A et al. (2004) Effect of potentially modifiable risk factors associated with myocardial infarction in 52 countries (the INTERHEART study): case-control study. Lancet 364: 937-952. doi:10.1016/S0140-6736(04)17018-9. PubMed: 15364185.
51. Elwood PC, Pickering JE, Fehily AM (2007) Milk and dairy consumption, diabetes and the metabolic syndrome: the Caerphilly prospective study. J Epidemiol Community Health 61: 695-698. doi:10.1136/jech.2006.053157. PubMed: 17630368.

52. Beydoun MA, Gary TL, Caballero BH, Lawrence RS, Cheskin LJ et al. (2008) Ethnic differences in dairy and related nutrient consumption among US adults and their association with obesity, central obesity, and the metabolic syndrome. Am J Clin Nutr 87: 1914-1925. PubMed: 18541585.
53. O'Neil CE, Nicklas TA, Liu Y, Franklin FA (2009) The impact of dairy product consumption on nutrient adequacy and weight of Head Start mothers. Public Health Nutr 12: 1693-1701. doi:10.1017/S1368980008003911. PubMed: 19000345.
54. Poddar KH, Hosig KW, Nickols-Richardson SM, Anderson ES, Herbert WG et al. (2009) Low-Fat Dairy Intake and Body Weight and Composition Changes in College Students. J Am Diet Assoc 109: 1433-1438. doi:10.1016/j.jada.2009.05.005. PubMed: 19631052.
55. Winkleby MA, Kraemer HC, Ahn DK, Varady AN (1998) Ethnic and socioeconomic differences in cardiovascular disease risk factors: findings for women from the Third National Health and Nutrition Examination Survey, 1988-1994. JAMA 280: 356-362. doi:10.1001/jama.280.4.356. PubMed: 9686553.
56. Chen M, Pan A, Malik VS, Hu FB (2012) Effects of dairy intake on body weight and fat: a meta-analysis of randomized controlled trials. Am J Clin Nutr 96: 735-747. doi:10.3945/ajcn.112.037119. PubMed: 22932282.
57. Ludwig DS, Willett WC (2013) Three Daily Servings of Reduced-Fat Milk: An Evidence-Based Recommendation? JAMA Pediatr: 1-2. PubMed: 23460048.
58. Conditions TNC (2013) CfC. Type 2 diabetes: the management of type 2 diabetes. . London: Royal College of Physicians.
59. Association AD (2013). Dairy: What Can I Eat?
60. Atkinson FS, Foster-Powell K, Brand-Miller JC (2008) International tables of glycemic index and glycemic load values: 2008. Diabetes Care 31: 2281-2283. doi:10.2337/dc08-1239. PubMed: 18835944.
61. Choi HK, Willett WC, Stampfer MJ, Rimm E, Hu FB (2005) Dairy consumption and risk of type 2 diabetes mellitus in men: a prospective study. Arch Intern Med 165: 997-1003. doi:10.1001/archinte.165.9.997. PubMed: 15883237.
62. Wheeler ML, Dunbar SA, Jaacks LM, Karmally W, Mayer-Davis EJ et al. (2012) Macronutrients, food groups, and eating patterns in the management of diabetes: a systematic review of the literature, 2010. Diabetes Care 35: 434-445. doi:10.2337/dc11-2216. PubMed: 22275443.
63. Services USDoHaH (2010) Dietary Guidelines for Americans 2010
64. Mozaffarian D, Micha R, Wallace S (2010) Effects on coronary heart disease of increasing polyunsaturated fat in place of saturated fat: a systematic review and meta-analysis of randomized controlled trials. PLOS Med 7: e1000252. PubMed: 20351774.
65. Truby H, Baic S, deLooy A, Fox KR, Livingstone MBE et al. (2006) Randomised controlled trial of four commercial weight loss programmes in the UK: initial findings from the BBC “diet trials”. BMJ 332: 1309-1314. doi:10.1136/bmj.38833.411204.80. PubMed: 16720619.
66. Bolland MJ, Avenell A, Baron JA, Grey A, MacLennan GS et al. (2010) Effect of calcium supplements on risk of myocardial infarction and cardiovascular events: meta-analysis. BMJ 341: c3691. doi:10.1136/bmj.c3691. PubMed: 20671013.
67. Benatar JR, Jones E, White H, Stewart RA (2013) A randomized trial evaluating the effects of change in dairy food consumption on cardio-metabolic risk factors. Eur J Prev Cardiol
68. Crichton GE, Howe PR, Buckley JD, Coates AM, Murphy KJ et al. (2012) Long-term dietary intervention trials: critical issues and challenges. Trials 13: 111. doi:10.1186/1745-6215-13-111. PubMed: 22812577.
