# Supplementary material for: Expression of Concern: Effects of High and Low Fat Dairy Food on Cardio-Metabolic Risk Factors: A Meta-Analysis of Randomized Studies
Source: PLoS One. 2023 Nov 13;18(11):e0283275. doi: 10.1371/journal.pone.0283275 (PMC10642843; doi:10.1371/journal.pone.0283275)
Supplement: S4 File — (DOCX) [file pone.0283275.s004.docx]

## Effects of high and low fat dairy food consumption on cardio-metabolic risk factors: A meta-analysis of randomised studies.

### Abstract

Importance: Clear guidelines on the health effects of dairy food are important given the high prevalence of obesity, cardiovascular disease and diabetes, and increasing global consumption of dairy food.

Objective**:** To evaluate the effects of increased dairy food in a healthy population on cardiometabolic risk factors.

Data Sources: Searches were performed until April 2013 using Medline, Science Direct, Embase, Google, the Cochrane Central Register of Controlled Trials, reference lists of articles, and proceedings of major meetings.

Study Selection: Healthy adults randomised to increased dairy food for more than one month without additional interventions.

Data Extraction and Synthesis**:** A standard list was used to extract descriptive, methodological and key variables from all eligible studies. If data was not included in the published report corresponding authors were contacted.

Results**:** 20 studies with 1677 participants with a median duration of dietary change of 26 (interquartile range 10-39) weeks and mean increase in dairy food intake of 3.6 (standard deviation 0.92) serves/day were included.

Increased dairy food intake was associated with a modest weight gain (+0.59, 95% confidence interval 0.34 to 0.84kg, p<0.0001) but no significant change in waist circumference (0.35 , -0.75 to 1.45 cm); insulin resistance (HOMA –IR -0.94 , -1.93 to 0.05 units); fasting glucose (0.87, -0.27 to 2.01 mg/dl); LDL-cholesterol (1.36 ,-2.38 to 5.09 mg/dl); HDL-cholesterol (0.45, -2.13 to 3.04 mg/dl); systolic (-0.13, -1.73 to 1.98 mmHg) and diastolic blood pressure (0.13, -1.73 to 1.98 mmHg) or C-reactive protein (-0.08, -0.63 to 0.48 mg/L). Results were similar for studies with low-fat and whole-fat dairy interventions.

Limitations: Most clinical trials were small and of modest quality.

Conclusion: Increasing whole fat and low fat dairy food consumption increases weight but has minor effects on other cardio-metabolic risk factors.

### Trial Registration ACTRN: ACTRN12613000401752

### Ethics approval number: NTX/10/11/115Introduction

Clear guidelines on the health effects of dairy food are important given the high and increasing prevalence of obesity, [^2^](#_ENREF_2) cardiovascular disease [^3^](#_ENREF_3) and diabetes [^4^](#_ENREF_4) in most countries, and the increasing global consumption of dairy food. [^5^](#_ENREF_5) Many current dietary guidelines promote low- fat dairy products as a healthy food. [^6^](#_ENREF_6)^,^ [^7^](#_ENREF_7) This advice is supported by observational studies which report that increased dairy food consumption is associated with lower blood pressure, [^8-12^](#_ENREF_8) weight reduction, [^13^](#_ENREF_13) improved insulin sensitivity, [^8^](#_ENREF_8)^,^ [^12^](#_ENREF_12)^,^ [^14^](#_ENREF_14)^,^ [^15^](#_ENREF_15) less inflammation [^16^](#_ENREF_16)^,^ [^17^](#_ENREF_17) and a lower ratio of total to HDL cholesterol. [^18^](#_ENREF_18) A modest inverse association between dairy consumption and cardiovascular disease has also been reported. [^19-21^](#_ENREF_19)

In contrast, whole fat dairy food is not recommended in most food guidelines [^22-24^](#_ENREF_22) because of the concern that saturated fat in dairy food may have an adverse effect on serum lipids which could increase the risk of cardiovascular disease. Despite these guidelines the effects of high fat dairy food on the risk of obesity, diabetes and cardiovascular disease are uncertain. A recent meta-analysis found no association between dietary saturated fat intake and the risk of cardiovascular disease. [^25^](#_ENREF_25) Whole fat dairy foods contain many fatty acids, which may have favourable as well as unfavourable effects on lipids and other cardio-metabolic risk factors. [^26^](#_ENREF_26) Also, the effects of reducing saturated fat from one food are determined by other dietary changes, including carbohydrates, and mono-unsaturated and poly-unsaturated fatty acids. [^18^](#_ENREF_18)

The effects of a high dairy food diet on diabetes and cardiovascular disease have not been evaluated in randomised clinical outcome trials. The large long term randomised dietary intervention studies which evaluated the ‘Dietary Approaches to Stop Hypertension’ (DASH) [^27^](#_ENREF_27) and ‘Mediterranean’ [^28^](#_ENREF_28) diets on clinical outcomes, while including increased low fat dairy food in the intervention, do not allow an for evaluation of the independent effects of changes in dairy food intake. Health effects of whole and low fat dairy food would be more reliably evaluated in clinical trials than in observational studies, and by assessing a number, rather than just one cardio-metabolic risk factor. We therefore undertook a meta-analysis of randomised clinical studies that evaluated effects of changing whole and low fat dairy food intake in healthy adults on a broad range of cardio-metabolic risk factors including weight, insulin resistance, lipids, blood pressure and c- reactive protein (CRP).

### Methods

We followed the PRISMA (<http://www.prisma-statement.Org>) guidelines throughout the design, implementation, analysis, and reporting of this meta-analysis. See Checklist S1 and File S1 for the PRISMA checklist and flow chart, respectively. A protocol for the study was designed (File S2) and the study was registered with the Australian New Zealand Clinical Trials Registry with trial registration number ACTRN12613000401752.

#### Search Strategy

We searched for all trials that randomised adults to increased dairy for at least one month without additional interventions (e.g. caloric restriction, multiple dietary interventions), had an appropriate control group, and sufficient data to calculate estimates of effect with standard deviations on at least one of the following: weight, waist circumference, blood pressure, HDL and LDL cholesterol, fasting glucose, insulin resistance and CRP. Studies were excluded if they were observational or otherwise non-randomised; were commentaries, reviews, or duplicate publications from the same study. We restricted inclusion to studies of healthy adults who did not have diabetes, hypertension or vascular disease. Both feeding and dietary advice trials and studies with a crossover or parallel group study design were included.

Searches were performed of literature published through March 2013 using Medline, Science Direct, Embase, Google, the Cochrane Central Register of Controlled Trials, reference lists of articles, and proceedings of major meetings for relevant literature. The search terms were ‘dairy’ and each of the following; ‘cardiometabolic risk’, ‘weight’, ‘waist circumference’, ‘glucose’, ‘insulin’, ‘insulin resistance’, ‘inflammation’, ‘inflammatory markers’, ‘blood pressure’, ‘cholesterol’ and ‘lipids’.

#### Assessment of study eligibility and data extraction

One reviewer screened all abstracts and titles and, upon retrieval of candidate studies, two team members (JB, KS) reviewed the full text to determine eligibility. If the study was eligible, data were abstracted by JB. Through an iterative process, a standard list was used to extract descriptive, methodological and key variables from all eligible studies. Data extracted included years the study was performed and reported, the primary aim of the study, population characteristics, funding source, control and intervention diets, duration of follow-up, estimates of effect and standard deviations. If data was not included in the published report corresponding authors were contacted. [^29^](#_ENREF_29)^,^ [^30^](#_ENREF_30) The quality of each study was rated using the Jadad score. [^31^](#_ENREF_31) Questions arising during data abstraction were resolved by discussion with all team members.

#### Definitions

Dairy food with less than1% fat, such as trim or low fat milk was categorized as a low- fat dairy food. Dairy food that included full-fat milk (3-4% fat), cheese, butter, cream and ice cream, was categorized as whole fat dairy food.

The method used to quantify insulin resistance was the homeostatic model assessment- Insulin resistance (HOMA-IR). [^32^](#_ENREF_32) This estimates steady state insulin sensitivity as units. The equation that is used is HOMA-IR = glucose (mmol/L) x insulin (munits/L) ÷22.5.

#### Statistical analysis

Each cardio-metabolic risk factor when on a higher and lower dairy food diet was compared between cases and controls from the same study. For those studies with 3 treatment groups, comparison was made between the control and the high dairy food group. Effects were measured at least 4 weeks after randomization, with the final results used for studies with more than one measurement during follow up. A negative effect size means that dairy food has a favourable effect on the cardiometabolic risk factor. Because an increase in HDL-cholesterol is considered beneficial, positive and negative exponents were switched to maintain consistency in presentation. In one study the standard deviation was not reported [^33^](#_ENREF_33) but calculated from the 95% confidence intervals.

For each cardio-metabolic risk factor, the weighted mean change from baseline to follow up was calculated across all included studies within each randomised group. The inverse-variance method, whereby study differences are weighted according to the reciprocal of their variance, was used to pool all standardized mean differences to yield an overall effect size with corresponding 95% confidence intervals.

When SD where not available, the mean weighted standard deviation of the group was used. For studies that used standard error of the mean (SEM) , this was converted to SD.

Each meta-analysis was assessed for heterogeneity by a Chi square test and *I^2^* statistic. A fixed effects model was used when heterogeneity was not present (*I^2^*=0) and a random effects model was used when statistical heterogeneity (I2≥1%) was present. A p-value of <0.05 was considered statistically significant. Studies are presented in Forrest plots in order of statistical power. Stratified analyses was studied by low-fat and whole- fat dairy, duration of dietary intervention (less than or greater than 6 months), body weight of study participants, and industry or public source of funding. Studies where the intervention was skim, trim or <1% dairy food are low fat dairy food studies. Sensitivity analyses were also conducted to evaluate the impact of selected studies on overall pooled estimates and heterogeneity. The Statistical analyses were performed using RevMan software version 5·2 (The Nordic Cochrane Centre, The Cochrane Collaboration, Copenhagen).

### Results

#### Search Results

The literature search yielded 5504 citations (2495 on Pub Med and 2849 on Science Direct, 160 on Google), which included 1844 duplicates. After title and abstract screening, the full text of 225 articles were evaluated, with 205 excluded either because the intervention included caloric restriction or had multiple dietary changes, or the study was in a population with disease (Figure 1). Twenty studies were included in the meta-analysis.

**Figure 1:** Study flow chart of the meta-analysis.


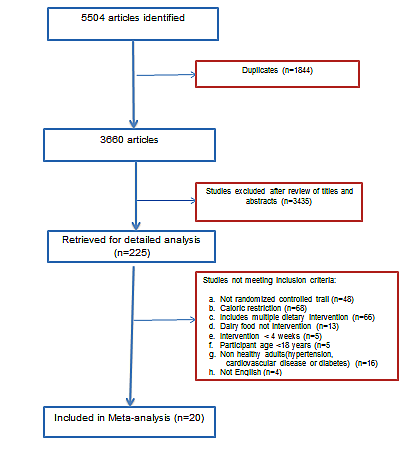


#### Characteristics of studies

Characteristics of the 20 included trials which included 1677 participants are summarized in Table 1. The average age was 51 (SD 16) years and 78% of participants were female. The median duration of follow up was 26 (IQR 10-39) weeks. The average difference in dairy food intake between groups was 3.12 (SD 0.62) standard serving sizes /day. Within studies there was no imbalance between randomised groups. One crossover study ^[29](#_ENREF_29)^ had a higher dropout rate for subjects initially randomised to low compared to high dairy food intake (49% vs. 22%).

Sixteen studies had a parallel group design and 4 were cross-over studies. In 10 studies increased dairy food included whole fat dairy food, while in another 10 studies, only low -fat dairy was advised. Fifteen of the studies were at least partly funded by the dairy or food industry.


Change in risk factors on a high and a lower dairy food diet in all studies combined are displayed in figures 2-10. Results stratified by duration of intervention, participant body weight and by funding source are displayed in Table 2.*Effects on body weight*

Eighteen [^11^](#_ENREF_11)^,^ [^29^](#_ENREF_29)^,^ [^30^](#_ENREF_30)^,^ [^33-47^](#_ENREF_33) studies reported effects on weight in 1629 individuals (Figure 2). The mean body mass index (BMI) at baseline was 25.6 (SD 6.2) kg/m^2^ and weight 77.7 (SD 16.2) kg. Increased dairy food intake was associated with a modest weight gain (+0.59, 95% confidence interval 0.34 to 0.84kg, p<0.0001). In six studies [^29^](#_ENREF_29)^,^ [^34^](#_ENREF_34)^,^ [^42-44^](#_ENREF_42)^,^ [^46^](#_ENREF_46) with 440 individuals, waist circumference did not change significantly (0.35, -0.75 to 0. 1.45cm) (Figure 3).

Weight gain was observed both in studies which increased low fat (+0.77, 0.32 to 1.21 kg, p<0.001) and whole fat dairy food (+0.44, 0.07 to 0.80kg, p=0.03). Modest weight gain was also observed in 10 studies (n= 692) which included overweight and obese subjects (+0.66, 0.04 to 1.29kg, p=0.04) and in 8 studies n= 937) of normal weight participants (+0.60, 0.35 to 0.85kg, p<0.001).

*Effects on insulin resistance*

In 8 studies, [^11^](#_ENREF_11)^,^ [^29^](#_ENREF_29)^,^ [^30^](#_ENREF_30)^,^ [^36^](#_ENREF_36)^,^ [^42^](#_ENREF_42)^,^ [^43^](#_ENREF_43)^,^ [^46^](#_ENREF_46)^,^ [^48^](#_ENREF_48) there was no significant change in fasting glucose on a higher compared to a lower dairy food diet (Figure 4). Four studies, [^42-44^](#_ENREF_42)^,^ [^46^](#_ENREF_46) assessed effects on HOMA-IR in 270 subjects (Figure 5). One [^43^](#_ENREF_43) did not report standard deviation so a weighted mean standard deviation from the other studies was used. HOMA-IR was recalculated in one study which used incorrect units. [^44^](#_ENREF_44) For all studies combined HOMA-IR was slightly improved on the high dairy food diet (-0.94, -1.93 to 0.05 units, p=0.06). However there was heterogeneity between studies (I^2^ =92%), accounted for by the two smallest studies [^42^](#_ENREF_42)^,^ [^43^](#_ENREF_43) which reported reduced insulin resistance on the high dairy food diet (-1.37, -1.64 to -1.10 units). HOMA-IR was similar on high and low dairy food diets in the two larger studies [^44^](#_ENREF_44)^,^ [^46^](#_ENREF_46) (-0.05, -0.26 to 0.17 units). HOMA-IR did not change significantly for studies stratified by body weight, duration of intervention or high versus low fat dairy food (Table 2).

*Effects on LDL and HDL cholesterol*

Eight studies [^11^](#_ENREF_11)^,^ [^29^](#_ENREF_29)^,^ [^30^](#_ENREF_30)^, [41,43, 44](#_ENREF_41),^ [^46^](#_ENREF_46)^,^ [^47^](#_ENREF_47) assessed effects on LDL- and/or HDL-cholesterol (n=664 for HDL, n=662 for LDL). For all studies combined there was no significant change in either LDL or HDL-cholesterol after increasing dairy food (Figures 6 and 7). Effects of HDL-c were consistent (I^2^=0%) across studies, but there was heterogeneity for LDL-c (I^2^=49%). There was no change in LDL cholesterol when whole fat dairy food (+6.18, 1.99 to 10.36 mg/dl]) or low fat dairy (-2.12,-3.98 to -0.26 mg/dl) food was increased (Figure 6). Results were similar for shorter and longer periods of dietary intervention and for studies which included normal and overweight or obese participants.

*Effects on C-reactive protein*

Five studies [^29^](#_ENREF_29)^,^ [^44^](#_ENREF_44)^,^ [^46^](#_ENREF_46)^,^ [^49^](#_ENREF_49)^,^ [^50^](#_ENREF_50) assessed effects on CRP in 400 individuals (Figure 8). For all studies combined there was no significant change in C-RP on a high dairy food diet (-0.08, -0.63 to 0.48 mg/dL). However, two smaller studies [^42^](#_ENREF_42)^,^ [^49^](#_ENREF_49) reported significant reductions in CRP with increased dairy food intake. There was no evidence for effects on CRP when studies were stratified by duration of dietary intervention, high and low fat dairy food, or normal or overweight subjects (Table 2).

*Effects on blood pressure*

Seven studies [^11^](#_ENREF_11)^,^ [^29^](#_ENREF_29)^,^ [^30^](#_ENREF_30)^,^ [^33^](#_ENREF_33)^,^ [^42^](#_ENREF_42)^,^ [^44^](#_ENREF_44)^,^ [^46^](#_ENREF_46)^,^ [^50^](#_ENREF_50) assessed effects on blood pressure in 711 participants (Figures 9 and 10). For all studies there was no significant change in either systolic blood pressure or diastolic blood pressure. There was also no evidence for effects on blood pressure when studies were stratified by duration of dietary intervention, high and low fat dairy food or for normal or overweight subjects.

*Evaluation of heterogeneity and sensitivity analysis*

Industry sponsored studies were more likely to report favourable effects on risk factors than non-industry sponsored studies (Table 2). Funnel plots identified that the study by Stancliffe [^42^](#_ENREF_42) reported decreases in LDL cholesterol, HOMA-IR, CRP and waist circumference on the increased dairy diet food beyond the 95% confidence range for all studies combined (see S2 File in the accompanying notice [68]). This study also reported the greatest decrease in blood pressure and weight of all studies. Excluding this study in a sensitivity analysis substantially decreased heterogeneity, but overall effects were similar, and there was no other consistent difference between smaller and larger studies. The study by Manios [^34^](#_ENREF_34) was the only study that fell outside the 95% confidence interval for weight, though excluding the study had no overall effect on results. This study had an imbalance in randomization with fewer people randomised to high dairy food (n=30) compared to the control group (n=40). The study by Ghadirian [^36^](#_ENREF_36) was outside the 95% confidence interval for fasting plasma glucose. In this study, the control group had a statistically significant reduction in fasting plasma glucose, with little change for the high dairy food group’. Repeated sensitivity analysis excluding this study showed no overall effect (+0.02,-0.05 to 0.09mmol/L, p=0.56) and a reduction in heterogeneity (25→0%). Results were also similar when analysis was repeated excluding the 4 cross-over studies.

On the basis of a funnel plot and Begg’s test, no significant publication bias was shown in the meta-analysis of body weight, waist circumference, insulin resistance, blood pressure, lipids and CRP.

### Discussion

This systematic analysis of randomised dietary intervention trials suggests that a moderate increase in dairy food consumption has no or small effect on the major cardiovascular and metabolic risk factors. [^51^](#_ENREF_51) This conclusion contrasts with results from several large epidemiological studies, which concluded that dairy food may have favourable effects on insulin resistance and decrease the risk of type 2 diabetes. [^8^](#_ENREF_8)^,^ [^52^](#_ENREF_52) However, in these observational studies [^33^](#_ENREF_33)^,^ [^53-55^](#_ENREF_53) dairy food intake was associated with an overall healthier eating pattern, healthier lifestyle, higher socio economic status and educational attainment, which are each associated with more favourable cardiometabolic profiles. [^56^](#_ENREF_56) Evaluating the effects of dairy food in randomised intervention trials, is likely to be more reliable than from observational studies, where associations may not be causal.

Several observational studies have suggested that dairy food may facilitate weight loss, particularly in obese and overweight individuals. [^13^](#_ENREF_13) Also in randomised trials where the intervention included both increased dairy food and caloric restriction, weight loss has been reported. [^57^](#_ENREF_57) However, in the current meta-analysis, which included studies which gave no advice on calorie restriction, increasing dairy food consumption resulted in a modest weight gain. Results were similar in studies which included overweight and obese participants. Whilst no direct comparison is possible, mean weight gain on low fat dairy food is double that of whole fat dairy food. This is counterintuitive but is in keeping with a recent viewpoint expressed in JAMA pediatrics [^58^](#_ENREF_58) which suggest that trim milk is associated with increased weight in children. It is likely that the weight gain was the result of increased total calories, in studies which encouraged greater dairy food intake without making other changes in diet. It is uncertain whether weight gain also occurs when dairy food is taken as part of, rather than in addition to a balanced diet.

Several diabetes guidelines [^6^](#_ENREF_6)^,^ [^59^](#_ENREF_59)^,^ [^60^](#_ENREF_60) recommend regular intake of low-fat dairy food because of its’ low glycaemic index. [^61^](#_ENREF_61) In observational studies [^52^](#_ENREF_52)^,^ [^62^](#_ENREF_62) persons in the highest quartile of dairy products consumption have less insulin resistance, and this association is strongest in those who are overweight or obese. In this analysis insulin sensitivity improved in two small studies [^42^](#_ENREF_42)^,^ [^43^](#_ENREF_43) with no demonstrated effect in the larger trials. [^44^](#_ENREF_44)^,^ [^46^](#_ENREF_46) In stratified analyses there was no effect in overweight and obese participants, or with whole or low fat dairy food interventions. Based on these observations, it is uncertain whether increasing dairy food improves insulin sensitivity, and further well designed studies are needed to resolve this question. A recent review by the American Diabetes Association [^63^](#_ENREF_63) concluded that ‘none of the components of dairy appear to have an effect on glycaemic control or cardiovascular disease risk reduction’, which is consistent with this analysis.

Many food guidelines encourage consumption of low- fat dairy food, but advise avoiding whole- fat dairy products. [^23^](#_ENREF_23)^,^ [^64^](#_ENREF_64) In the current analysis LDL-c did not change significantly when whole-fat dairy food consumption was increased. Whilst the risk of cardiovascular disease is reduced when saturated fats are replaced by unsaturated fats, [^65^](#_ENREF_65) the reasons for this may be multifactorial. This study suggests that effects on LDL-cholesterol may not the primary reason.

Dairy food intake was associated with lower blood pressure in observational studies, [^9^](#_ENREF_9)^,^ [^14^](#_ENREF_14) and in the large randomised DASH study. [^27^](#_ENREF_27) In the DASH study the intervention included increased low fat dairy food, reduced total and saturated fat, and increased fruit and vegetables. In a secondary analysis it was estimated that low-fat dairy food could account for about half of the observed 5.5mmHg decrease in systolic blood pressure. However it is not possible to reliably estimate the effects of each dietary component when the intervention includes multiple dietary changes. For this reason the DASH study and studies of the Mediterranean diet [^28^](#_ENREF_28) were not included in this meta-analysis. In this meta-analysis, the confidence intervals exclude significant effects (>1.6mmHg) of increasing dairy food on systolic and diastolic blood pressure.

*Limitations of meta-analysis*

The majority of subjects included in the meta-analysis were women, but there is currently no evidence for different effects of diet by gender. The diverse population and age range of subjects included makes the results relevant to improving lifestyle risk factors for diabetes and cardiovascular disease in the general population. Studying healthy populations also avoids possible treatment and disease effects on the outcomes of interest. Further research is needed to confirm similar neutral effects of dairy food in patients with established diabetes and cardiovascular disease.

It is possible that the duration of the dietary intervention was not long enough to identify effects on risk factors, but stratified analyses suggest similar results for longer and shorter periods of dietary intervention. The increase of 3.6 servings each day is a substantial dietary change, and previous studies suggest dietary interventions influence risk factors within one month. [^27^](#_ENREF_27)^,^ [^66^](#_ENREF_66)

Most studies included were relatively small. It is difficult to blind diet studies and the level of compliance with the dietary interventions was often uncertain. Several studies reported significant adverse or favourable effects of increasing dairy food on one or more risk factors, but sensitivity analyses suggested these studies had only a small effect on overall estimates. It is also possible that smaller studies which found no effects have not been published. Three quarters of the studies were funded by the dairy or food industry, and results were more favourable for industry compared to non-industry funded studies

.

This meta-analysis stratified studies by ‘low fat’ and ‘whole fat’ interventions, but a direct comparison of these studies may not be reliable, and no trials, which directly compare ‘low’ with ‘whole’ fat dairy food diets, have been reported. Because studies were small, and some may be unreliable, the analysis cannot exclude a small increase in LDL-c, with increase in whole-fat dairy food consumption. Studies are also needed to evaluate the effects of other components of dairy food. To provide a better estimate of health effects the meta-analysis evaluated associations with multiple rather than just one or two risk factors. However dairy food could influence the risk of cardiovascular disease or diabetes by pathways other than the risk factors measured. [^67^](#_ENREF_67) The influence of dairy food on clinical outcomes rather than risk factors is most important for dietary guidelines. However, currently, no completed randomised trials allow independent assessment of the effects of changing dairy food on diabetic complications or cardiovascular events.

#### Conclusion

Increase in both whole and low fat dairy food, without other dietary interventions, is associated with a modest weight gain, with no or minor effects on other cardio-metabolic risk factors. These observations suggest that for most healthy individuals it is reasonable to include both low and whole fat dairy food as part of a healthy diet.

1. Benatar JR, Sidhu K, Stewart RAH. Effects of high and low fat dairy food on cardio-metabolic risk factors: A meta-analysis of randomized studies. *PloS one*. 2013;8:e76480

2. Swinburn BA, Sacks G, Hall KD, McPherson K, Finegood DT, Moodie ML, Gortmaker SL. The global obesity pandemic: Shaped by global drivers and local environments. *The Lancet*. 2011;378:804-814

3. Organisation WH. The atlas of heart disease and stroke. 2012;2013

4. Federation ID. Idf diabetes atlas. 2011;2013

5. Development; TOfEC-oa. Oecd-fao agricultural outlook 2011-2020. 2011

6. American Diabetes A, Bantle JP, Wylie-Rosett J, Albright AL, Apovian CM, Clark NG, Franz MJ, Hoogwerf BJ, Lichtenstein AH, Mayer-Davis E, Mooradian AD, Wheeler ML. Nutrition recommendations and interventions for diabetes: A position statement of the american diabetes association. *Diabetes care*. 2008;31 Suppl 1:S61-78

7. United States Department of Agriculture. Us choosemyplate. 2012;2012

8. Pereira MA, Jacobs DR, Jr., Van Horn L, Slattery ML, Kartashov AI, Ludwig DS. Dairy consumption, obesity, and the insulin resistance syndrome in young adults: The cardia study. *JAMA : the journal of the American Medical Association*. 2002;287:2081-2089

9. Toledo E, Delgado-Rodriguez M, Estruch R, Salas-Salvado J, Corella D, Gomez-Gracia E, Fiol M, Lamuela-Raventos RM, Schroder H, Aros F, Ros E, Ruiz-Gutierrez V, Lapetra J, Conde-Herrera M, Saez G, Vinyoles E, Martinez-Gonzalez MA. Low-fat dairy products and blood pressure: Follow-up of 2290 older persons at high cardiovascular risk participating in the predimed study. *The British journal of nutrition*. 2009;101:59-67

10. Engberink MF, Geleijnse JM, de Jong N, Smit HA, Kok FJ, Verschuren WM. Dairy intake, blood pressure, and incident hypertension in a general dutch population. *The Journal of nutrition*. 2009;139:582-587

11. van Meijl LE, Mensink RP. Low-fat dairy consumption reduces systolic blood pressure, but does not improve other metabolic risk parameters in overweight and obese subjects. *Nutrition, metabolism, and cardiovascular diseases : NMCD*. 2011;21:355-361

12. Lutsey PL, Steffen LM, Stevens J. Dietary intake and the development of the metabolic syndrome: The atherosclerosis risk in communities study. *Circulation*. 2008;117:754-761

13. Abargouei AS, Janghorbani M, Salehi-Marzijarani M, Esmaillzadeh A. Effect of dairy consumption on weight and body composition in adults: A systematic review and meta-analysis of randomized controlled clinical trials. *International journal of obesity*. 2012

14. Liu S, Song Y, Ford ES, Manson JE, Buring JE, Ridker PM. Dietary calcium, vitamin d, and the prevalence of metabolic syndrome in middle-aged and older u.S. Women. *Diabetes care*. 2005;28:2926-2932

15. Snijder MB, van der Heijden AA, van Dam RM, Stehouwer CD, Hiddink GJ, Nijpels G, Heine RJ, Bouter LM, Dekker JM. Is higher dairy consumption associated with lower body weight and fewer metabolic disturbances? The hoorn study. *The American journal of clinical nutrition*. 2007;85:989-995

16. Panagiotakos DB, Pitsavos CH, Zampelas AD, Chrysohoou CA, Stefanadis CI. Dairy products consumption is associated with decreased levels of inflammatory markers related to cardiovascular disease in apparently healthy adults: The attica study. *J Am Coll Nutr*. 2010;29:357-364

17. Esmaillzadeh A, Azadbakht L. Dairy consumption and circulating levels of inflammatory markers among iranian women. *Public health nutrition*. 2010;13:1395-1402

18. Mensink RP, Zock PL, Kester AD, Katan MB. Effects of dietary fatty acids and carbohydrates on the ratio of serum total to hdl cholesterol and on serum lipids and apolipoproteins: A meta-analysis of 60 controlled trials. *The American journal of clinical nutrition*. 2003;77:1146-1155

19. Hu FB, Stampfer MJ, Manson JE, Ascherio A, Colditz GA, Speizer FE, Hennekens CH, Willett WC. Dietary saturated fats and their food sources in relation to the risk of coronary heart disease in women. *The American journal of clinical nutrition*. 1999;70:1001-1008

20. Kelemen LE, Kushi LH, Jacobs DR, Jr., Cerhan JR. Associations of dietary protein with disease and mortality in a prospective study of postmenopausal women. *American journal of epidemiology*. 2005;161:239-249

21. Elwood PC, Strain JJ, Robson PJ, Fehily AM, Hughes J, Pickering J, Ness A. Milk consumption, stroke, and heart attack risk: Evidence from the caerphilly cohort of older men. *Journal of epidemiology and community health*. 2005;59:502-505

22. European Dietary Guidelines. European dietary guidelines.2012

23. American Heart Association Nutrition Committee, Lichtenstein AH, Appel LJ, Brands M, Carnethon M, Daniels S, Franch HA, Franklin B, Kris-Etherton P, Harris WS, Howard B, Karanja N, Lefevre M, Rudel L, Sacks F, Van Horn L, Winston M, Wylie-Rosett J. Diet and lifestyle recommendations revision 2006: A scientific statement from the american heart association nutrition committee. *Circulation*. 2006;114:82-96

24. Australian Dietary Recommendations. Australian dietary recommendations. 2012;2012

25. Siri-Tarino PW, Sun Q, Hu FB, Krauss RM. Meta-analysis of prospective cohort studies evaluating the association of saturated fat with cardiovascular disease. *The American journal of clinical nutrition*. 2010;91:535-546

26. Iggman D RU. Role of different dietary saturated fatty acids for cardiometabolic risk. *Clinical Lipidology*. 2011;6:209-223.

27. Appel LJ, Moore TJ, Obarzanek E, Vollmer WM, Svetkey LP, Sacks FM, Bray GA, Vogt TM, Cutler JA, Windhauser MM, Lin PH, Karanja N. A clinical trial of the effects of dietary patterns on blood pressure. Dash collaborative research group. *The New England journal of medicine*. 1997;336:1117-1124

28. Estruch R, Ros E, Salas-Salvado J, Covas MI, Pharm D, Corella D, Aros F, Gomez-Gracia E, Ruiz-Gutierrez V, Fiol M, Lapetra J, Lamuela-Raventos RM, Serra-Majem L, Pinto X, Basora J, Munoz MA, Sorli JV, Martinez JA, Martinez-Gonzalez MA, the PSI. Primary prevention of cardiovascular disease with a mediterranean diet. *The New England journal of medicine*. 2013:1279-1290

29. Crichton GE, PR CH, Buckley JD, Coates AM, Murphy KJ. Dairy consumption and cardiometabolic health: Outcomes of a 12-month crossover trial. *Nutrition & metabolism*. 2012;9:19

30. Barr SI, McCarron DA, Heaney RP, Dawson-Hughes B, Berga SL, Stern JS, Oparil S. Effects of increased consumption of fluid milk on energy and nutrient intake, body weight, and cardiovascular risk factors in healthy older adults. *J Am Diet Assoc*. 2000;100:810-817

31. Jadad AR MR, Caroll D. Assessing the quality of reports of randomized clinical trials: Is blinding necessary? *Controlled Clin. Trials* 1996;17:1-12

32. Levy JC, Matthews DR, Hermans MP. Correct homeostasis model assessment (homa) evaluation uses the computer program. *Diabetes care*. 1998;21:2191-2192

33. Alonso A, Zozaya C, Vazquez Z, Alfredo Martinez J, Martinez-Gonzalez MA. The effect of low-fat versus whole-fat dairy product intake on blood pressure and weight in young normotensive adults. *Journal of human nutrition and dietetics : the official journal of the British Dietetic Association*. 2009;22:336-342

34. Manios Y, Moschonis G, Koutsikas K, Papoutsou S, Petraki I, Bellou E, Naoumi A, Kostea S, Tanagra S. Changes in body composition following a dietary and lifestyle intervention trial: The postmenopausal health study. *Maturitas*. 2009;62:58-65

35. Kukuljan S, Nowson CA, Bass SL, Sanders K, Nicholson GC, Seibel MJ, Salmon J, Daly RM. Effects of a multi-component exercise program and calcium-vitamin-d3-fortified milk on bone mineral density in older men: A randomised controlled trial. *Osteoporosis international : a journal established as result of cooperation between the European Foundation for Osteoporosis and the National Osteoporosis Foundation of the USA*. 2009;20:1241-1251

36. Ghadirian P, Shatenstein B, Verdy M, Hamet P. The influence of dairy products on plasma uric acid in women. *European journal of epidemiology*. 1995;11:275-281

37. Chee WS, Suriah AR, Chan SP, Zaitun Y, Chan YM. The effect of milk supplementation on bone mineral density in postmenopausal chinese women in malaysia. *Osteoporosis international : a journal established as result of cooperation between the European Foundation for Osteoporosis and the National Osteoporosis Foundation of the USA*. 2003;14:828-834

38. Eagan MS, Lyle RM, Gunther CW, Peacock M, Teegarden D. Effect of 1-year dairy product intervention on fat mass in young women: 6-month follow-up. *Obesity*. 2006;14:2242-2248

39. Gunther CW, Legowski PA, Lyle RM, McCabe GP, Eagan MS, Peacock M, Teegarden D. Dairy products do not lead to alterations in body weight or fat mass in young women in a 1-y intervention. *The American journal of clinical nutrition*. 2005;81:751-756

40. Lau EM, Woo J, Lam V, Hong A. Milk supplementation of the diet of postmenopausal chinese women on a low calcium intake retards bone loss. *Journal of bone and mineral research : the official journal of the American Society for Bone and Mineral Research*. 2001;16:1704-1709

41. Palacios C, Bertran JJ, Rios RE, Soltero S. No effects of low and high consumption of dairy products and calcium supplements on body composition and serum lipids in puerto rican obese adults. *Nutrition*. 2011;27:520-525

42. Stancliffe RA, Thorpe T, Zemel MB. Dairy attentuates oxidative and inflammatory stress in metabolic syndrome. *The American journal of clinical nutrition*. 2011;94:422-430

43. Tardy AL, Lambert-Porcheron S, Malpuech-Brugere C, Giraudet C, Rigaudiere JP, Laillet B, Leruyet P, Peyraud JL, Boirie Y, Laville M, Michalski MC, Chardigny JM, Morio B. Dairy and industrial sources of trans fat do not impair peripheral insulin sensitivity in overweight women. *The American journal of clinical nutrition*. 2009;90:88-94

44. Wennersberg MH, Smedman A, Turpeinen AM, Retterstol K, Tengblad S, Lipre E, Aro A, Mutanen P, Seljeflot I, Basu S, Pedersen JI, Mutanen M, Vessby B. Dairy products and metabolic effects in overweight men and women: Results from a 6-mo intervention study. *The American journal of clinical nutrition*. 2009;90:960-968

45. Zemel MB, Richards J, Milstead A, Campbell P. Effects of calcium and dairy on body composition and weight loss in african-american adults. *Obesity research*. 2005;13:1218-1225

46. Benatar JR, Jones E, White HD, Stewart RAH. A randomized trial evaluating the effects of change in dairy food consumption on cardio-metabolic risk factors. *European journal of preventive cardiology*. 2013

47. Baran D, Sorensen A, Grimes J, Lew R, Karellas A, Johnson B, Roche J. Dietary modification with dairy products for preventing vertebral bone loss in premenopausal women: A three-year prospective study. *The Journal of clinical endocrinology and metabolism*. 1990;70:264-270

48. Gardner CD, Messina M, Kiazand A, Morris JL, Franke AA. Effect of two types of soy milk and dairy milk on plasma lipids in hypercholesterolemic adults: A randomized trial. *J Am Coll Nutr*. 2007;26:669-677

49. Zemel MB, Sun X, Sobhani T, Wilson B. Effects of dairy compared with soy on oxidative and inflammatory stress in overweight and obese subjects. *The American journal of clinical nutrition*. 2010;91:16-22

50. van Meijl LE, Mensink RP. Effects of low-fat dairy consumption on markers of low-grade systemic inflammation and endothelial function in overweight and obese subjects: An intervention study. *The British journal of nutrition*. 2010;104:1523-1527

51. Yusuf S, Hawken S, Ounpuu S, Dans T, Avezum A, Lanas F, McQueen M, Budaj A, Pais P, Varigos J, Lisheng L, Investigators IS. Effect of potentially modifiable risk factors associated with myocardial infarction in 52 countries (the interheart study): Case-control study. *Lancet*. 2004;364:937-952

52. Elwood PC, Pickering JE, Fehily AM. Milk and dairy consumption, diabetes and the metabolic syndrome: The caerphilly prospective study. *Journal of epidemiology and community health*. 2007;61:695-698

53. Beydoun MA, Gary TL, Caballero BH, Lawrence RS, Cheskin LJ, Wang Y. Ethnic differences in dairy and related nutrient consumption among us adults and their association with obesity, central obesity, and the metabolic syndrome. *The American journal of clinical nutrition*. 2008;87:1914-1925

54. O'Neil CE, Nicklas TA, Liu Y, Franklin FA. The impact of dairy product consumption on nutrient adequacy and weight of head start mothers. *Public health nutrition*. 2009;12:1693-1701

55. Poddar KH, Hosig KW, Nickols-Richardson SM, Anderson ES, Herbert WG, Duncan SE. Low-fat dairy intake and body weight and composition changes in college students. *Journal of the American Dietetic Association*. 2009;109:1433-1438

56. Winkleby MA, Kraemer HC, Ahn DK, Varady AN. Ethnic and socioeconomic differences in cardiovascular disease risk factors: Findings for women from the third national health and nutrition examination survey, 1988-1994. *JAMA : the journal of the American Medical Association*. 1998;280:356-362

57. Chen M, Pan A, Malik VS, Hu FB. Effects of dairy intake on body weight and fat: A meta-analysis of randomized controlled trials. *The American journal of clinical nutrition*. 2012;96:735-747

58. Ludwig DS, Willett WC. Three daily servings of reduced-fat milk: An evidence-based recommendation? *JAMA pediatrics*. 2013:1-2

59. Conditions. TNCCfC. Type 2 diabetes: The management of type 2 diabetes. . 2013;2013

60. American Diabetes Association. Dairy. *What Can I Eat?* 2013;2013

61. Atkinson FS, Foster-Powell K, Brand-Miller JC. International tables of glycemic index and glycemic load values: 2008. *Diabetes care*. 2008;31:2281-2283

62. Choi HK, Willett WC, Stampfer MJ, Rimm E, Hu FB. Dairy consumption and risk of type 2 diabetes mellitus in men: A prospective study. *Arch Intern Med*. 2005;165:997-1003

63. Wheeler ML, Dunbar SA, Jaacks LM, Karmally W, Mayer-Davis EJ, Wylie-Rosett J, Yancy WS, Jr. Macronutrients, food groups, and eating patterns in the management of diabetes: A systematic review of the literature, 2010. *Diabetes care*. 2012;35:434-445

64. Services USDoHaH. Dietary guidelines for americans 2010. 2010

65. Mozaffarian D, Micha R, Wallace S. Effects on coronary heart disease of increasing polyunsaturated fat in place of saturated fat: A systematic review and meta-analysis of randomized controlled trials. *PLoS medicine*. 2010;7:e1000252

66. Truby H, Baic S, deLooy A, Fox KR, Livingstone MBE, Logan CM, Macdonald IA, Morgan LM, Taylor MA, Millward DJ. Randomised controlled trial of four commercial weight loss programmes in the uk: Initial findings from the bbc “diet trials”. *Bmj*. 2006;332:1309-1314

67. Bolland MJ, Avenell A, Baron JA, Grey A, MacLennan GS, Gamble GD, Reid IR. Effect of calcium supplements on risk of myocardial infarction and cardiovascular events: Meta-analysis. *Bmj*. 2010;341:c3691

1. The PLOS ONE Editors. (2023) Expression of Concern: Effects of High and Low Fat Dairy Food on Cardio-Metabolic Risk Factors: A Meta-Analysis of Randomized Studies. PLOS ONE.
